# Targeting *Pseudomonas aeruginosa* quorum sensing with sodium salicylate modulates immune responses *in vitro* and *in vivo*

**DOI:** 10.3389/fcimb.2023.1183959

**Published:** 2023-08-08

**Authors:** Erik Gerner, Paula Milena Giraldo-Osorno, Anna Johansson Loo, Rininta Firdaus, Heithem Ben Amara, Maria Werthén, Anders Palmquist, Peter Thomsen, Omar Omar, Sofia Almqvist, Margarita Trobos

**Affiliations:** ^1^Department of Biomaterials, Institute of Clinical Sciences, The Sahlgrenska Academy, University of Gothenburg, Gothenburg, Sweden; ^2^Centre for Antibiotic Resistance Research in Gothenburg (CARe), Gothenburg, Sweden; ^3^Mölnlycke Health Care AB, Gothenburg, Sweden; ^4^Department of Biomedical Dental Sciences, College of Dentistry, Imam Abdulrahman Bin Faisal University, Dammam, Saudi Arabia

**Keywords:** *Pseudomonas aeruginosa*, sodium salicylate, quorum sensing, immune response, wound infection, biomaterial-associated infection (BAI), inflammation, phagocytosis

## Abstract

**Introduction:**

Chronic infections are a major clinical challenge in hard-to-heal wounds and implanted devices. *Pseudomonas aeruginosa* is a common causative pathogen that produces numerous virulence factors. Due to the increasing problem of antibiotic resistance, new alternative treatment strategies are needed. Quorum sensing (QS) is a bacterial communication system that regulates virulence and dampens inflammation, promoting bacterial survival. QS inhibition is a potent strategy to reduce bacterial virulence and alleviate the negative impact on host immune response.

**Aim:**

This study investigates how secreted factors from *P. aeruginosa* PAO1, cultured in the presence or absence of the QS inhibitor sodium salicylate (NaSa), influence host immune response.

**Material and methods:**

*In vitro*, THP-1 macrophages and neutrophil-like HL-60 cells were used. *In vivo*, discs of titanium were implanted in a subcutaneous rat model with local administration of *P. aeruginosa* culture supernatants. The host immune response to virulence factors contained in culture supernatants (+/-NaSa) was characterized through cell viability, migration, phagocytosis, gene expression, cytokine secretion, and histology.

**Results:**

*In vitro*, *P. aeruginosa* supernatants from NaSa-containing cultures significantly increased THP-1 phagocytosis and HL-60 cell migration compared with untreated supernatants (-NaSa). Stimulation with NaSa-treated supernatants *in vivo* resulted in: (i) significantly increased immune cell infiltration and cell attachment to titanium discs; (ii) increased gene expression of IL-8, IL-10, ARG1, and iNOS, and (iii) increased GRO-α protein secretion and decreased IL-1β, IL-6, and IL-1α secretion, as compared with untreated supernatants.

**Conclusion:**

In conclusion, treating *P. aeruginosa* with NaSa reduces the production of virulence factors and modulates major immune events, such as promoting phagocytosis and cell migration, and decreasing the secretion of several pro-inflammatory cytokines.

## 1 Introduction

Chronic infections, such as hard-to-heal wounds and biomaterial-associated infections, lead to prolonged patient suffering and have a considerable socioeconomic burden (Romling et al., 2014). A common feature of chronic infections is the presence of biofilms, i.e., aggregated bacteria embedded in a self-produced three-dimensional matrix of extracellular polymeric substances (EPS) (Flemming and Wingender, 2010). Biofilms are substantially more tolerant to host immune responses and antimicrobials compared with their planktonic counterparts (Stewart, 2015; Zaborowska et al., 2017). It has been estimated that 80% of chronic wounds contain biofilms (Malone et al., 2017), suggesting their substantial contribution to the delayed wound healing (Schultz et al., 2017). Biomaterials have an increased risk of infection since the presence of a biomaterial decreases by 10 000-fold the minimum number of bacteria required to cause infection (Ribeiro et al., 2012; Zimmerli and Trampuz, 2013). Contaminating bacteria can form biofilms on the implant surface and in the surrounding tissue (Broekhuizen et al., 2007; Svensson et al., 2015), and patients suffering infections caused by strong biofilm-producing strains show a significantly increased risk of recurrent infection (Svensson Malchau et al., 2021). Implant-associated infections commonly result in implant removal and prolonged antimicrobial therapy. Current therapies are based on antibiotics, but the global threat of antibiotic-resistant infections, projected to surpass cancer as a leading cause of death by 2050 and exceed $300 billion in annual costs worldwide, highlights the need to explore alternative antimicrobial treatment strategies (Sugden et al., 2016; Dadgostar, 2019).

One potential alternative to fight antibiotic resistant infections is an anti-virulence approach by inhibiting quorum sensing (QS), a bacterial communication system dependent on cell and signal density that regulates bacterial virulence (Azimi et al., 2020). *Pseudomonas aeruginosa*, an opportunistic gram-negative pathogen with a well-characterized QS system, causes a wide variety of acute and chronic infections, including wound infections (Lyczak et al., 2000) and biomaterial-associated infections (Hsieh et al., 2009; Cerioli et al., 2020). Given its multidrug resistance and high virulence, *P. aeruginosa* is listed by the World Health Organization as a global health threat (Mancuso et al., 2021). The broad arsenal of QS-regulated virulence factors in *P. aeruginosa*, including tissue-degrading enzymes, toxins and biofilm-related factors (Behzadi et al., 2021; Chadha et al., 2022b), has led to extensive research focusing on identifying effective quorum sensing inhibitors (QSIs) (Chadha et al., 2022a). In addition, *P. aeruginosa* QS signals and virulence factors have been shown to have immunomodulatory effects that are involved in the pathogen’s ability to escape the host immune response and thus ensure its survival (Jiajie et al., 2020). While QS signals of *P. aeruginosa* have been shown to have pro-inflammatory properties when administered to immune cells, their effect in combination with bacterial stimuli has also demonstrated anti-inflammatory properties (Jiajie et al., 2020). The latter effect includes attenuation of inflammasome activation and the subsequent production of interleukin 1 beta (IL-1β) (Yang et al., 2017), degradation of inflammatory mediators (Leidal et al., 2003), interleukin 10 (IL-10) induction (Glucksam-Galnoy et al., 2013) and increased apoptosis (Tateda et al., 2003). The strategy of inhibiting bacterial QS, e.g., by administration of small signal-interfering molecules, thus has the potential to reduce the infectious capabilities of bacteria, including *P. aeruginosa*, while regaining the protective effects of the immune system.

An anti-virulence-based treatment that prevents biofilm formation, damaging virulence factors, and excessive inflammation, particularly in chronic wounds and biomaterial-associated infections, would offer some advantages compared to traditional antibiotic therapy. Antibiotics primarily aim to kill or inhibit the growth of bacteria (Kohanski et al., 2010). In contrast, QSIs reduce bacterial virulence without compromising bacterial viability or growth (Hentzer et al., 2003). This approach reduces the selective pressure for resistance development in bacteria, potentially mitigating the emergence of antimicrobial resistant strains. Moreover, while antibiotics do not directly modulate the host immune response, QS inhibition can attenuate excessive inflammation associated with *P. aeruginosa* infections (Tang et al., 2022). This modulation of the immune response can help prevent tissue damage caused by an exaggerated immune reaction. Furthermore, *P. aeruginosa* biofilms are notoriously difficult to eradicate with antibiotics alone. QS inhibition disrupts the communication between bacterial cells within the biofilm, potentially making them more susceptible to clearance by the immune system or other antimicrobial interventions (Brackman et al., 2011). An anti-virulence approach based on QS inhibition may offer a potential solution for addressing chronic infections with a biofilm pathogenesis.

Despite extensive *in vitro*, *in vivo*, and to some extent clinical research (Smyth et al., 2010; Van Delden et al., 2012), no treatments based on QS-inhibition are yet available. This is, at least partly, related to the lack of appropriate understanding of the biological mechanisms governing the regulation of the immune response to the targeted microorganisms that have been exposed to QS-inhibiting substances. Our research group has previously shown that sodium salicylate (NaSa), the sodium salt of the bioactive metabolite of acetylsalicylic acid (Aspirin®), interferes with *P. aeruginosa* QS, reducing virulence factor and biofilm production (Gerner et al., 2020; Gerner et al., 2021). In this study, we aim to evaluate the effect of NaSa on the ability of *P. aeruginosa* to modulate immune response processes, such as phagocytosis, immune cell migration, and secretion of cytokines, chemokines, and tissue-degrading enzymes. This study is based on *in vitro* models, using THP-1 macrophages and neutrophil-like HL-60 cells, as well as an *in vivo* rat soft tissue model.

## 2 Materials and methods

### 2.1 Bacterial supernatant preparation

**Figure 1:**
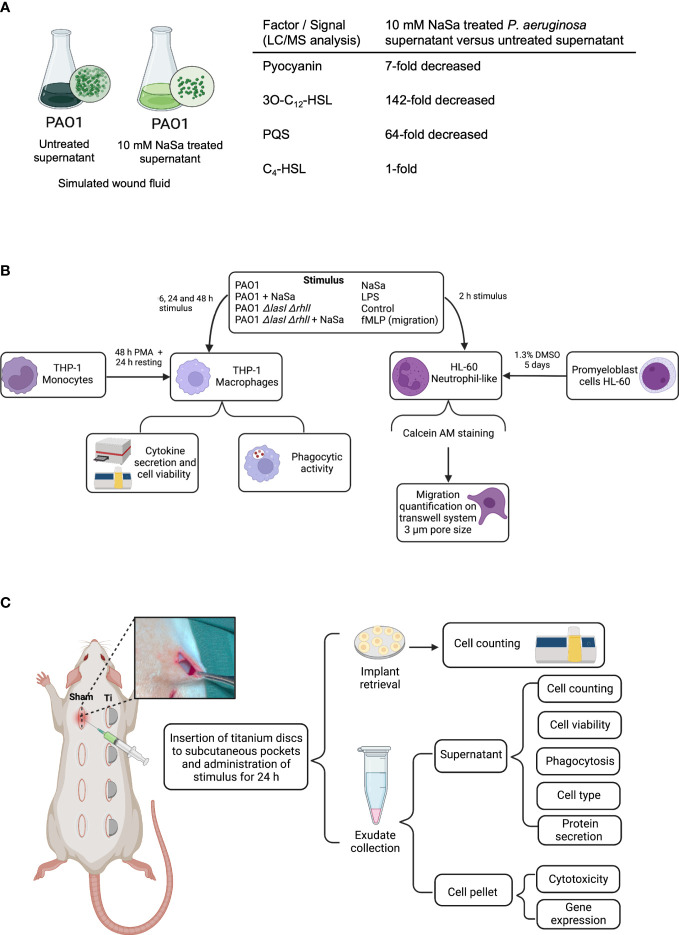
Study design. **(A)** Schematic view of bacterial supernatant preparation. Liquid chromatography mass spectrometry (LC/MS) concentrations of selected virulence factor and signals in undiluted supernatants, presented as fold-change differences between NaSa-treated *P. aeruginosa* PAO1 cultures versus untreated cultures. **(B)** *In vitro* and **(C)** *in vivo* experimental flowchart. These illustrations were created with BioRender.com.

Bacterial supernatants were generated from cultures of *P. aeruginosa* PAO1 wild-type (*Pseudomonas* genetic stock center; strain PAO0001) and *P. aeruginosa* PAO1 *ΔlasIΔrhlI* (Hentzer et al., 2003). Colonies grown overnight on 5% horse blood Columbia agar plates (Medium Department, Clinical Microbiology Lab, Sahlgrenska University Hospital, Gothenburg, Sweden) were collected and diluted to an optical density (OD_546_) of 0.1 (10^8^ colony-forming units (CFU)/mL) in 0.9% saline with 50% non-heat inactivated foetal bovine serum (FBS) (HyClone, GE Healthcare Life Sciences, Chicago, Illinois, USA) and further diluted 1:10 in the same media supplemented with 0 or 10 mM NaSa (ReagentPlus®, purity ≥99.5%, Sigma Aldrich, Munich, Germany). One mL (10^7^ CFU/mL) was then added to each well of a 48-well plate (Nunc, Thermo Fisher Scientific, Roskilde, Denmark) to allow for static biofilm formation for 48 h at 35°C in a humidified incubator. The medium containing the secreted factors was centrifuged at 4 000 g for 10 min, and the supernatants were filtered sterilized through 0.2 µM filters (Sartorius Stedim Biotech, Göttingen, Germany), aliquoted and frozen at –20°C until further use (**Figure 1A**).

### 2.2 QS signal and pyocyanin quantification

Pyocyanin, *N*-(3-oxododecanoyl)-L-homoserine lactone (3-oxo-C_12_-HSL), *N*-butanoyl-L-homoserine lactone (C_4_-HSL) and 2-heptyl-3-hydroxy-4-quinolone (PQS) (all from Sigma Aldrich, Munich, Germany) were diluted to 5 mg/mL in dimethyl sulfoxide (DMSO) to prepare standard mixtures of all analytes (5 μg/mL each) in 50% FBS in saline and calibration solutions of 0.025, 0.125, 0.249, 0.375 and 0.625 μg/mL. The supernatants of *P. aeruginosa* wild-type (WT) grown with and without NaSa, prepared as described above, were acidified by the dropwise addition of 12 M HCl to pH=2 and left at room temperature overnight. Cold acetonitrile, 800 μL, was mixed with 200 µL of sample. The resulting mixture was centrifuged at 17 000 g for 30 min at 15°C and the supernatants were collected.

A Single Quadrupole liquid chromatography mass spectrometry (LC/MS) (Agilent Technologies, Santa Clara, California, USA) equipped with an Agilent InfinityLab Poroshell 120EC-C18 analytical column (4.6×100 mm, 2.7 µm) and an Agilent InfinityLab Poroshell 120 EC-C18 guard column (4.6×5 mm, 2.7 µm) was used for chromatographic separation of the analytes. The mobile phase consisted of 10 mM ammonium formate aqueous solution with 0.1% of formic acid (A) and 10 mM ammonium formate methanol solution with 0.1% of formic acid (B). The gradient elution was run as follows: 0–2.5 min, 25% B; 2.5–7.0 min, 65% B; 7.0–9.0 min, 100% B; 9.0–9.10 min, 25% B; 9.10–12 min, 25% B. The mobile phase flow rate was set at 0.8 mL/min, the column temperature was 50°C and the injection volume was 1 μL. The mass spectrometer, equipped with electrospray ionization (ESI) as the ion source, was operated in positive ion mode. Both SCAN and SIM modes were used. The m/z values and fragmentor voltage were set as follows: C_4 = _172 ([M+H]+), 80 V; C_12 = _298 ([M+H]+), 140 V; Pyocyanin=211 ([M+H]+), 135 V; PQS=260 ([M+H]+), 135 V. All samples were analyzed in triplicate.

### 2.3 *In vitro* models

A schematic of the experimental procedures *in vitro* is presented in **Figure 1B**.

#### 2.3.1 Stimulation of human monocytes

The human monocytic cell line THP-1 (ATCC, Manassas, USA) was grown in Roswell Park Memorial Institute (RPMI) 1640 medium supplemented with 10% FBS, 0.5% β-mercaptoethanol (Sigma Aldrich, Munich, Germany) and 1% penicillin/streptomycin (PEST) solution (Gibco Life Technologies, Carlsbad, California, USA) in a 37°C humidified incubator with 5% CO_2_. Cells between passages 6 and 9 were used. For viability and protein secretion studies, THP-1 monocytes were seeded at a density of 250 000 cells/mL in Nunc 24-well plates (Thermo Fisher Scientific) in 1 mL media as explained above. Cells were then stimulated with 10 ng/mL phorbol-12-myristate-13-acetate (PMA, Sigma Aldrich, Munich, Germany) for 48 h to induce macrophage differentiation, followed by 24 h of resting time in fresh media without PMA. For phagocytosis studies, the same differentiation protocol was applied, but cells were seeded in black 384-well microtiter plates with clear bottoms (Greiner Bio-One, Kremsmünster, Austria) at a density of 40 000 cells/well.

For viability and protein secretion studies, differentiated and resting macrophage cells were stimulated with 20% and 1% supernatants (final concentrations) of PAO1 WT and PAO1 *ΔlasIΔrhlI* cultures with and without NaSa. LPS (Sigma Aldrich, Munich, Germany, *Escherichia coli* O111:B4) at 10 ng/mL, RPMI media with 2 mM NaSa, RPMI media with 0.1 mM NaSa, and RPMI media alone were used as controls. For phagocytosis experiments, cells were stimulated with 5% and 10% (final concentrations) of PAO1 WT supernatants from bacterial cultures grown with and without 10 mM NaSa. RPMI media alone and media supplemented with 1 mM NaSa were used as controls.

Cell viability, protein secretion, and phagocytosis were assessed after 6, 24, and 48 h of stimulation. Three independent experiments were performed, with three technical replicates for cell viability and protein secretion experiments and seven technical replicates for phagocytosis experiments.

#### 2.3.2 Cytokine secretion and viable cell counting

After cell stimulation for 6, 24 and 48 h, media for protein secretion analysis were collected in 1.5 mL Eppendorf tubes. To evaluate the number of viable cells, the wells were gently washed twice with PBS to remove nonadherent nonviable cells before the addition of NucleoCounter^®^ lysis and stabilization buffer (200 + 200 µL). Lysed samples were loaded in a Nucleocassette™ precoated with fluorescent propidium iodide, which stains cell nuclei, and the results were then quantified using a NucleoCounter^®^ NC-200 (ChemoMetec A/S, Allerod, Denmark).

The collected media was centrifuged at 300 g for 5 min, and the supernatants were aliquoted and stored at −80°C until analysis of the concentrations of the pro-inflammatory cytokines interleukin 1-beta (IL-1β), interleukin-12 (IL-12), interleukin-6 (IL-6), and tumor necrosis factor-alpha (TNF-α); the anti-inflammatory cytokines tumor necrosis factor receptor I (TNF-RI), tumor necrosis factor receptor II (TNFR-II), interleukin-10 (IL-10), and interleukin-1 receptor antagonist (IL-1Ra); the chemokines interleukin-8 (IL-8), monocyte chemoattractant protein 1 (MCP-1), macrophage inflammatory protein 1-alpha (MIP-1α/CCL3), growth-regulated protein alpha (GRO-α/CXCL-1); the antibacterial cytokines IFN-γ-inducible protein alpha (IP-10/CXCL-10) and macrophage migration inhibitory factor (MIF); and the tissue-degrading enzymes matrix metalloproteinase-1 (MMP-1), matrix metalloproteinase-2 (MMP-2), matrix metalloproteinase-3 (MMP-3), matrix metalloproteinase-7 (MMP-7), matrix metalloproteinase-9 (MMP-9), and matrix metalloproteinase-13 (MMP-13) using enzyme-linked immunosorbent assay (ELISA)-based multiplexed chemiluminescent analysis (Quansys Biosciences, Logan, Utah, USA) according to the manufacturer’s instructions. The detection of luminescent units or pixel intensity units was performed using the Q-View^TM^ Imager LS with Q-View software, and the results were converted to pg/mL using defined protein standards. Three separate experiments were performed, each with three technical replicates.

#### 2.3.3 NF-κB activation

THP-1-blue nuclear factor kappa B (NF-κB) cells (InvivoGen, Toulouse, France) with an NF-κB inducible alkaline protease reporter construct were used to investigate the effect of NaSa on LPS-induced NF-κB activity. The reporter cells were propagated in RPMI supplemented with 10% FBS and 100 µg/mL normocin and differentiated into macrophages as described previously, using a cell seeding density of 100 000 cells per well in 96-well plates (Nunc, Roskilde, Denmark). Next, RPMI media was replaced and supplemented with 0.5 ng/mL LPS and 0-10 mM NaSa, and RPMI media without LPS or NaSa were used as control. After 6 and 24 h incubation at 37°C, media was collected and mixed in a 1:10 ratio with Quanti-blue solution (Invivogen, Toulouse, France) in a new 96-well plate. After 45 min of incubation at 37°C, the OD at 655 nm was measured with a plate reader (BMG LABTECH, Ortenberg, Germany) as an indirect measurement of NF-κB activity. Cell numbers, assessed using a NucleoCounter^®^, were used to normalize the OD data.

#### 2.3.4 Phagocytosis

The assessment of phagocytosis was performed using pH-rodo™ red *Staphylococcus aureus* bioparticles (Thermo Fisher Scientific, Roskilde, Denmark), which allow the visualization and quantification of ingested bioparticles. The bioparticles are nonfluorescent at neutral pH outside of the cell, but as they are internalized in endosomes that fuse with lysosomes, the pH gradually decreases, and their fluorescence increases.

After 6, 24 and 48 h of stimulation with bacterial supernatants and controls, as described previously, the media was discarded, and 50 µL pH-rodo BioParticles solution in Live Cell Imaging Solution (Invitrogen, Waltham, Massachusetts, USA) was added. Following a 2 h incubation at 37°C, fluorescence was measured at 544/590 nm (ex/em) using a plate reader. Next, cell viability was analyzed by adding 50 µL of 1 µM calcein AM to each well. Calcein AM is hydrolyzed intracellularly by esterases in viable cells, producing calcein, a strongly fluorescent compound. After 30 min of incubation at 37°C, fluorescence was measured at 490/520 nm (ex/em). The fluorescence intensity from the pH-rodo bioparticles was normalized to calcein fluorescence to account for the cytotoxic effects of the different stimuli. Three separate experiments were performed, each with seven technical replicates.

#### 2.3.5 Cell migration

Cell migration was assessed in a Fluoroblok 96-well transwell system (Corning, Corning, New York, USA). The human promyeloblast cell line HL-60, ATCC-CCL-240 (LGC Standards, Wesel, Germany) was grown in RPMI media supplemented with 10% FBS (Sigma Aldrich, Munich, Germany) and 1% PEST (Gibco) in a 37°C humidified incubator with 5% CO_2_. The cell concentration was adjusted to 10^6^ cells/mL in the same media supplemented with 1.3% DMSO and incubated for five days at 37°C for differentiation into neutrophil-like cells (Hauert et al., 2002). The cells were labeled with 1 µM calcein AM for 15 min, followed by three centrifugation steps at 200 g for 5 min to wash away any unbound dye. The pellet was resuspended in serum-free RPMI at 4 x 10^6^ cells/mL. Fifty microliters of cell suspension were added to 96-well filter compartments with a 3 µM pore size. As stimuli in the receiver plate, 225 µL of 10% and 20% PAO1 WT and PAO1 *ΔlasIΔrhlI* supernatant from cultures +/- 10 mM NaSa was added to each well. As controls, 1 and 2 mM NaSa, 10 nM N-formylmethionyl-leucyl-phenylalanine (fMLP) (Sigma Aldrich, Munich, Germany) and RPMI alone were used; all stimuli were diluted in serum-free RPMI. The filter plate was attached to the receiver plate and placed in a microplate reader at 37°C. Bottom-read fluorescence was measured at 490/520 nm (ex/em) every 5 min for 1 h as an indirect measurement of the number of cells migrating through the membrane. Three independent experiments were performed, each with two technical replicates.

### 2.4 *In vivo* soft tissue model

A schematic of the experimental procedures *in vivo* is presented in **Figure 1C**.

#### 2.4.1 Soft tissue infection procedure

Thirty-two male Sprague-Dawley rats (200–300 g) fed with a standard pellet diet and water were used in the study, which was approved by the Local Ethical Committee for Laboratory Animals (Gothenburg, Sweden) (Dnr 1091/17). After a minimum of seven days of acclimatization at the animal facility, the rats were anesthetized by inhalation of 4% isoflurane (airflow of 650 mL/min), shaved on the dorsal side, disinfected with 5 mg/mL chlorhexidine (Fresenius Kabi, Bad Homburg, Germany) and maintained anesthetized using 2% isoflurane (airflow 450 mL/min via a mask; Univentor 400 anesthesia unit (Univentor, Zejtun, Malta). Eight incisions were made on the parasagittal plane followed by blunt dissection to create subcutaneous pockets into which titanium discs were inserted [9 mm diameter and 2.2 mm thick machined disks (Christers Finmekaniska Ab, Skövde, Sweden) of titanium grade 4 (Zapp Medical Alloys GmbH, Ratingen, Germany)]. In each animal, four implants were inserted into four pockets while the remaining pockets did not receive implants (sham), and to account for body side variability (left *vs* right) implants and sham sites were rotated according to a pre-established scheme. Eight rats were used per group. Into implanted and non-implanted (sham) pockets were added 50 µL of undiluted *P. aeruginosa* WT supernatant from biofilm cultures grown in a 1:1 mix of saline and FBS with and without 10 mM NaSa (group I and II). In animals serving as controls, 50 µL of a 1:1 mix of FBS and saline (diluent control, group III) or 10 mM NaSa (NaSa control, group IV) were added into the pockets. The incisions were closed with three simple interrupted sutures (Monocryl 4-0 FS-2, Eticon LLC, Johnson & Johnson, United Kingdom). Analgesic was administered subcutaneously to each rat directly after surgery and 8 h postsurgery (0.03 mg/kg Temgesic®, Reckitt Benckiser, Slough, UK). After 24 h, the rats were anesthetized by inhalation of 4% isoflurane and sacrificed by an overdose intraperitoneal injection of 60 g/L pentobarbital (APL, Stockholm, Sweden). All sutures were removed, and the implants were retrieved. Exudates were obtained from all the pockets by flushing 5 times with 300 µL ice-cold HBSS (Gibco) and kept on ice. Exudates from the implanted sites or sham sites were pooled before the division of the exudate for the different analyses.

#### 2.4.2 Cell numbers, viability and types

The quantification of host cells adhering to the implants and in the exudates was performed using the NucleoCounter^®^ system. For implants, total cell counts were quantified, while for exudates, viability was assessed by analyzing both dead and total cell counts. Cell toxicity was further analyzed by quantifying the lactate dehydrogenase (LDH) content in the supernatants of centrifuged exudates (300 g, 5 min) using an LDH-activity kit according to the manufacturer’s instructions (Sigma Aldrich, Munich, Germany).

To determine the cell types in the exudates, 50 000 cells from the exudate were applied onto a microscopic slide using cytospin centrifugation (Shandon Southern Products, Runcorn, United Kingdom). The cells were stained with May-Grünewald Giemsa, and the percentages of polymorphonuclear (PMN) and mononuclear cells were determined by counting at least 200 cells per sample using a Nikon Eclipse E600 light microscope (Nikon, Minato, Japan).

#### 2.4.3 Histology

Soft tissue samples were dissected (following implant removal in titanium-implanted sites) and fixed in formalin, dehydrated, and infiltrated in paraffin. Sections were cut at a thickness of ∼5 μm (Leica RM 2255, Leica Biosystems Nussloch GmbH, Nussloch, Germany), deparaffinized in xylene, and stained with hematoxylin and eosin. Histological analysis was carried out under an optical microscope (Nikon Eclipse E600, Nikon, Minato, Japan). Micrographs were acquired with a Plan Apo 20x/0.5 objective and a Plan Apo 40x/0.75 objective.

To quantify the density of cells infiltrating the soft tissues, five micrographs were obtained at 20x-magnification from each histological section (n = 5−6 per group) at the bottom tissue interfacing with the surgical pockets (**Supplementary Figures S1A, B**). Images were then imported into Qupath software (Bankhead et al., 2017) to semi-automatically detect cell nuclei stained with hematoxylin. The number of detections per tissue area was calculated.

#### 2.4.4 Phagocytosis

Phagocytic activity of collected immune cells was evaluated using the pH-rodo-based technique described above. Fifty thousand viable cells diluted in live imaging solution were added to clear bottom black 96-well plates (Corning) prior to the addition of 50 µL pH-rodo *S. aureus* bioparticles. After 2 h of incubation at 37°C, the fluorescence intensity was measured using a plate reader. Depending on the number of cells retrieved, three to five technical replicates were included.

#### 2.4.5 Cytokines

Concentrations of cytokines (interferon γ (IFN-γ), interleukin 1α (IL-1α), IL-1β, IL-6, IL-12, and TNF-α) in the supernatant fraction of the exudates were analyzed using a rat multiplexing ELISA kit (Quansys Biosciences, Logan, Utah, USA) according to the manufacturer’s instructions. Additional single-cytokine ELISA kits (Abbexa Ltd, Cambridge, United Kingdom) were used to analyze GRO-α, MCP-1, IL-8, and plasminogen activator inhibitor-1 (PAI-1) according to the manufacturer’s instructions.

#### 2.4.6 Gene expression

Gene expression of proinflammatory cytokines, interleukin 18 (IL-18), IL-1β and TNF-α; anti-inflammatory cytokine IL-10; growth factors, vascular endothelial growth factor (VEGF) and transforming growth factor β1 (TGF-β1); coagulation factors, tissue factor (TF) and PAI-1/SERPINE1; scavenger receptor, cluster of differentiation (CD163); chemokines, GRO-α/CXCL1, IL-8 and MCP-1; M1 phenotype marker, nitric oxide synthase (iNOS) and M2 phenotype marker, arginase 1 (ARG1) from exudates from sham and titanium-implanted sites were analyzed by quantitative reverse transcription polymerase chain reaction (RT-qPCR) (all primers were purchased from Bio‐Rad Laboratories, CA, USA). The cell pellet was resuspended in 200 µL of DNA/RNA Shield (Zymo Research, Irvine, California, US) and frozen at -80°C. RNA isolation was achieved using an RNeasy mini kit (Qiagen, Hilden, Germany) according to the manufacturer’s instructions. The isolated RNA was frozen at -80°C. Thereafter, total RNA was reverse transcribed into cDNA using TATAA grandScript cDNA synthesis kit (TATAA Biocenter AB, Gothenburg, Sweden) in 10 µL reactions. Pooled RNA within each group was used as the no reverse transcriptase control. The cDNA was diluted to 90 µL RNase-free H_2_O (Thermo‐Scientific, USA) and frozen at -20°C. Diluted samples were mixed with Bio-Rad SYBR green (Bio‐Rad Laboratories, CA, USA) and primers in 10 μL reactions in duplicate on the CFX96 platform (Bio‐Rad Laboratories, CA, USA). Moreover, before quantitative polymerase chain reaction (qPCR) analysis, a reference panel was screened. The software GeneEx (MultiD Analyses AB, Gothenburg, Sweden) was used to determine the best stable reference gene for normalization. Based on the analysis, the most stably expressed reference gene was YHWAZ. Gene expression levels were normalized using the relative comparative ΔΔCq method.

### 2.5 Statistics

All results are presented as the mean ± standard deviation. The normality of distribution was tested using the Shapiro–Wilk normality test. Multiple group comparisons were performed using one-way ANOVA with Bonferroni adjustment for normally distributed data and the Kruskal-Wallis H test for nonnormally distributed data. Related groups were analyzed using Wilcoxon’s matched pair test. The data were statistically evaluated in Graphpad Prism 9 (Dotmatics, Boston, Massachusetts, USA) using a significance level of 95%.

## 3 Results

### 3.1 Inhibition of QS signalling

LC/MS was performed to characterize the bacterial stimuli and to confirm the QS-inhibitory effect of NaSa, targeting three QS signals and the QS-regulated virulence factor pyocyanin. In undiluted supernatants, the concentrations of pyocyanin, 3-oxo-C_12_-HSL, and PQS in the NaSa-treated *P. aeruginosa* supernatants were 7-, 142-, and 64-fold lower, respectively, than those in the untreated supernatants (**Figure 1A**). The concentration of the QS signal C_4_-HSL was unaffected. These findings suggest that NaSa treatment effectively reduces the levels of QS signals and pyocyanin, supporting the QS-inhibitory effect of NaSa and dampening of virulence in *P. aeruginosa*.

### 3.2 *In vitro* host response to virulence factors from NaSa-treated *Pseudomonas aeruginosa*

#### 3.2.1 *Pseudomonas aeruginosa* supernatant is cytotoxic to human macrophages

**Figure 2:**
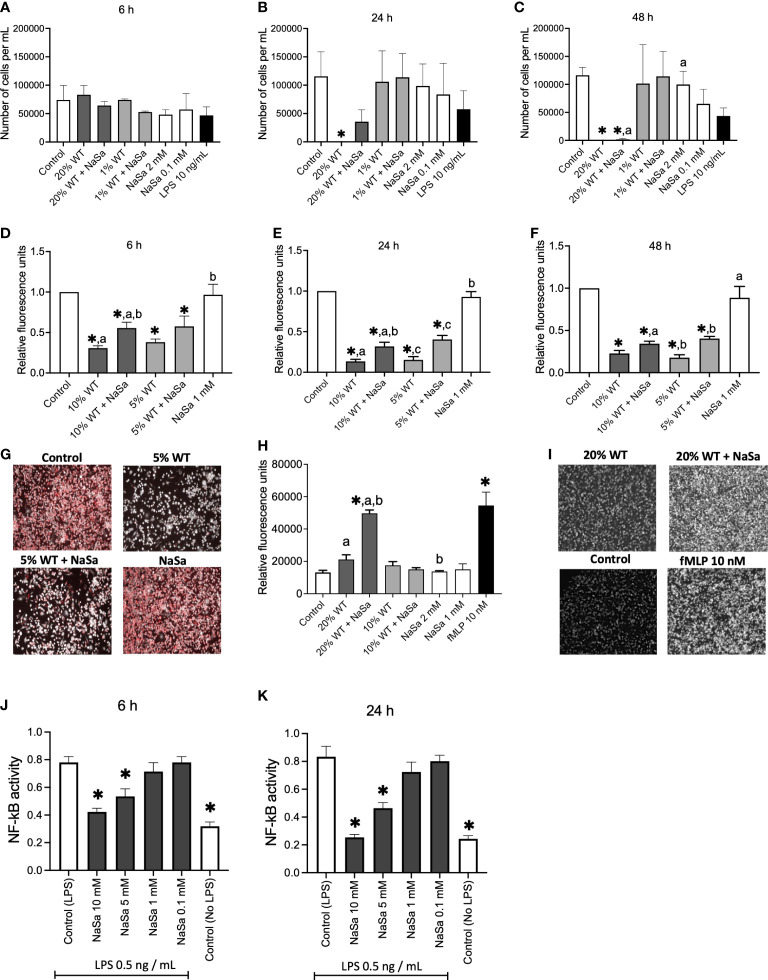
*Pseudomonas aeruginosa* untreated and treated with NaSa modulates immune responses in THP-1 macrophages and HL-60 neutrophil-like cells. **(A–C)** Number of viable THP-1 macrophages following incubation with *P. aeruginosa* supernatant (20% and 1%) treated ± NaSa for **(A)** 6 h, **(B)** 24 h and **(C)** 48 h analyzed using NucleoCounter^®^ (n=3, with 3 technical replicates). **(D–F)** Phagocytosis activity (pH-rodo fluorescence) normalized by cell viability of THP-1 macrophages stimulated during **(D)** 6, **(E)** 24 and **(F)** 48 h with 5% and 10% supernatant from cultures ± NaSa. (n=3, with 7 technical replicates). **(G)** Example of red fluorescence micrographs of pH-rodo *Staphylococcus aureus* bioparticles. **(H)** Neutrophil-like HL-60 cell migration towards *P. aeruginosa* supernatants (20% and 10%) from cultures ± NaSa, fMLP is used as a positive migration stimulus. **(I)** Example of fluorescence micrographs of HL-60 cell in receiver compartments. **(J, K)** NF-κB activity of THP-1 macrophages stimulated with 0.5 ng/mL LPS and 0.1-10 mM NaSa during **(J)** 6 h and **(K)** 24 h. (n=3, with 3 technical replicates). Significant differences between treatments are indicated by letters, where bars that share the same letters are significantly different (*p* < 0.05). Data represent the mean, and error bars the standard deviation. (*) significant compared to the media control (*p* < 0.05). Different supernatant concentrations have been used in the different assays due to viability differences between cell types.

To determine if the reduced secretion of virulence factors, after QS-inhibition with NaSa, decreased cytotoxicity, the viability of THP-1 macrophages was assessed. Stimulation with 1% supernatants did not result in any cytotoxicity (**Figures 2A–C**; **Supplementary Figures S2A–C**). The group of THP-1 macrophages stimulated with supernatants from *P. aeruginosa* had a similar number of viable cells compared with the RPMI media control after 6 h (**Figure 2A**; **Supplementary Figure S2A**). After 24 h, while the addition of 20% WT supernatant resulted in complete cell death, the 20% NaSa-treated WT supernatant showed 30% cell viability compared with the control (**Figure 2B**). No viable cells were observed with either untreated or NaSa-treated 20% WT supernatants at 48 h (**Figure 2C**). Viability was similar to control for cells subjected to the 20% supernatants from the *ΔlasIΔrhlI* QS mutant strain (with reduced virulence) at 24 h (**Supplementary Figure S2B**), except for a slight decrease in the viability by the untreated 20% mutant supernatant at 48 h (**Supplementary Figure S2C**). The presence of NaSa, at concentrations equivalent to those found in supernatants (0.1 or 2 mM), did not impact the cell viability (**Figures 2A–C**; **Supplementary Figures S2A–C**). In conclusion, the factors contained in *P. aeruginosa* supernatants were cytotoxic for THP-1 macrophages, whereas slightly less cytotoxic in case of NaSa-treatment at 24 h. The NaSa doses contained in the supernatants did not appear to induce evident cytotoxic effects.

#### 3.2.2 *Pseudomonas aeruginosa* NaSa-treated supernatant increases phagocytosis activity in THP-1 macrophages compared with untreated supernatant

Phagocytosis is a central defense mechanism against infection. Therefore, we investigated if the decreased virulence by NaSa is also associated with an improved phagocytic ability of macrophages to ingest *S. aureus* bioparticles. In general, stimulation of THP-1 macrophages with diluted WT supernatants from *P. aeruginosa* cultures (at a concentration of 10% or 5%), reduced the phagocytosis of pH-rodo red *S. aureus* bioparticles by 40-90% as compared with the RPMI media control; this effect was seen with NaSa-treated and untreated supernatants (**Figures 2D–F**). NaSa-treated diluted supernatants (10% or 5%) resulted in increased phagocytosis compared to corresponding supernatants without NaSa; relative increases ranged from 80-140% after 6-24 h and 130% after 48 h. In contrast, exposure of THP-1 macrophages to 1 mM NaSa alone did not affect their phagocytic ability as compared with the control. Representative micrographs for selected groups are shown in **Figure 2G**. Taken together, these results indicated that the reduced virulence activity of NaSa is associated with an improved phagocytic function of THP-1 macrophages.

#### 3.2.3 *Pseudomonas aeruginosa* supernatant treated with NaSa stimulates migration of neutrophil-like differentiated HL-60 cells

Quorum sensing signals and associated virulence factors may affect recruitment of immune cells to the site of infection. Here, we investigated the potential differences in neutrophil chemotaxis towards untreated and NaSa-treated *P. aeruginosa* supernatant. In a transwell migration system with a pore size of 3 µm, the number of HL-60 cells that migrated toward the 20% NaSa-treated WT supernatant was 2.3-fold higher than the number that migrated toward the corresponding 20% untreated supernatant (**Figure 2H**). The opposite trend was observed for the 20% mutant supernatant, where there was a slight decrease in cell migration (by ~25%) with NaSa treatment (**Supplementary Figure S2D**). NaSa alone did not have any effect on HL-60 cell migration. Representative micrographs of the receiver compartment for selected groups after 1 h of migration are shown in **Figure 2I**, and they indicate an increased chemotaxis of HL-60 cells stimulated with NaSa-treated supernatant and the chemokine fMLP, as compared to control and untreated supernatant. Pilot experiments with undifferentiated cells resulted in fluorescence readings in the range of 0-10% compared with those for differentiated cells (data not shown), indicating that the differentiation protocol was effective. Labelling of HL-60 cells using calcein AM resulted in a fluorescent signal that directly correlated to the cell number (R^2^ = 0.99) with a lower detection limit of 7 000 cells per well (**Supplementary Figures S2E, F**). In summary, NaSa-treated supernatants resulted in an increased neutrophil cell recruitment in contrast to cells subjected to untreated supernatant.

#### 3.2.4 NaSa treatment of *Pseudomonas aeruginosa* supernatant modulates the inflammatory response of THP-1 macrophages

**Figure 3:**
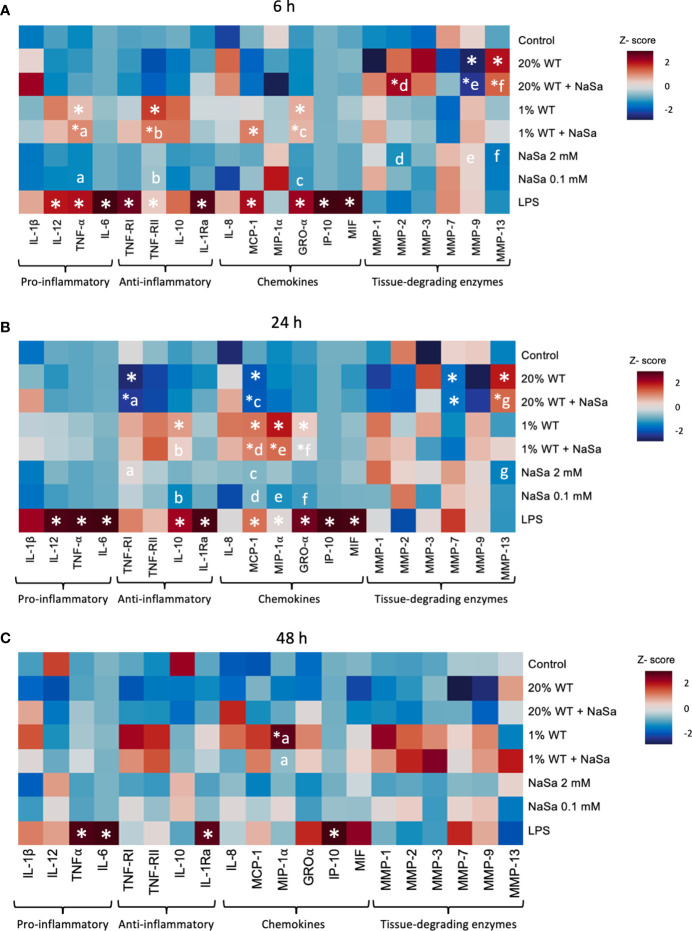
Effect of *Pseudomonas aeruginosa* supernatant from cultures with and without NaSa on the immune response of THP-1 macrophages. Levels of cytokines after stimulation of THP-1 macrophages with *P. aeruginosa* supernatant cultured ± NaSa quantified by multiplex ELISA using Q-Plex™ Array during **(A)** 6 h, **(B)** 24 h and **(C)** 48 h. Cytokine levels were standardized by calculating a Z-score (n=3, with 3 technical replicates). Significant differences between treatments are indicated by letters, where proteins that share the same letters are significantly different (*p* < 0.05). (*) significant compared to the media control.

*P. aeruginosa* is known to produce immunomodulatory factors involved in immune evasion. Here we evaluated the effect of NaSa treatment of *P. aeruginosa* on the inflammatory cell response by measuring NF-κB activity and key cytokines and chemokines produced by macrophages. First, we evaluated the effect of NaSa, at a relevant range of concentrations, on NF-κB activity (**Figures 2J, K**). Compared with the RPMI media control, NF-κB activity in LPS-treated THP-1 macrophages was reduced by 31-46% and 44-70% after 6 and 24 h, respectively, in the presence of 5-10 mM NaSa (**Figures 2J, K**). There was no change with 0.1 or 1 mM NaSa. Considering all experimental groups, LPS treatment alone was generally associated with the highest levels of cytokine secretions (**Figures 3A–C**). Generally, the concentration-dependent effects of the 1% and 20% supernatants varied between cytokines and time points. The levels of most cytokines (MCP-1, IL-10, GRO-α, TNF-α, TNF-RI, TNF-RII, MMP-1, and MMP-9) were higher in the 1% WT supernatant group whereas MMP-13 was higher in the 20% WT supernatant group. Some cytokines (IL-6, IP-10, and IL-12) had low or undetectable levels. Only LPS resulted in high levels of secretion of cytokines IL-6 and IL-10. There were no observable effects of NaSa treatment alone on cytokine secretion compared with the media control (**Figures 3A–C**).

Irrespective of NaSa treatment, the cytokine data suggested that supernatants from the mutant strain were more potent in promoting cytokine secretion than corresponding WT supernatants. At 6 h, TNF-α, IL-1Ra, MCP-1, GRO-α, and MMP-9 levels were 13.6-, 2.3-, 48-, 305-, and 6.1-fold higher, respectively, with the 20% mutant supernatant than the 20% WT supernatant (**Supplementary Figures S3A**, **S4**). The opposite was observed for MMP-13, which was increased 6-fold upon stimulation by the 20% WT supernatant compared to the 20% mutant supernatant. At the 6 h time point, the levels of GRO-α were 1.4-fold higher in the untreated 20% mutant supernatant group than in the corresponding NaSa-treated supernatant group. At 24 h, the levels of TNF-α, TNF-RI, TNF-RII, IL-10, MCP-1, MIP-1α, GRO-α, and MMP-9 were at least 4-fold higher upon stimulation with the 20% mutant supernatant than the 20% WT supernatant (**Supplementary Figures S3B**, **S4**). TNF-α and GRO-α levels were 4.5 and 2.5-fold higher, respectively, upon stimulation with the 1% mutant supernatant compared with the 1% WT supernatant, while MIP-1α levels were 2.5-fold lower. At 48 h, the levels of TNF-RII, MMP-1, MMP-7, and MMP-9 were up to 14-fold higher with the 20% mutant supernatant than the 20% WT supernatant (**Supplementary Figures S3C**, **S4**). Collectively, upon LPS-stimulation, NaSa treatment reduced the activity of the NF-κB transcription factor. Nonetheless, NaSa-treated supernatant did not significantly alter the secretion levels of the analysed cytokines as compared to the untreated supernatant. The mutant strain resulted in a stronger inflammatory response compared to the WT strain, however this could partially be attributed to the significantly decreased cell viability caused by the WT strain compared to the mutant.

### 3.3 *In vivo* host response to virulence factors from NaSa-treated *Pseudomonas aeruginosa*

**Figure 4:**
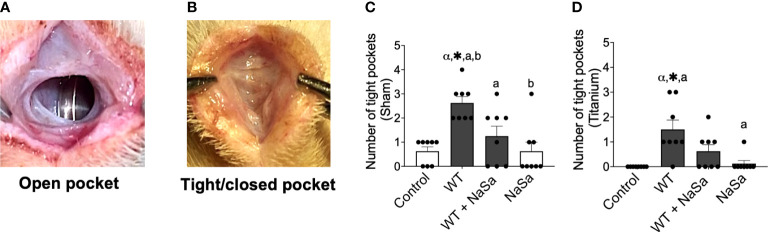
Tissue response after supernatant administration from *Pseudomonas aeruginosa* cultures with and without NaSa in a soft-tissue rat infection model. **(A)** Open pockets after 24 h in control animals and **(B)** tight/closed pockets after 24 h in *P. aeruginosa* supernatant-stimulated animals. **(C, D)** Number of tight pockets per group in **(C)** sham and **(D)** titanium sites. Significant differences between treatments are indicated by letters, where bars that share the same letters are significantly different (*p* < 0.05), n=8. (*) significant compared to the control. (α) significant between sham and titanium using Wilcoxon signed-rank test (*p* < 0.05).

Taking into account the above *in vitro* results demonstrating that NaSa treatment of *P. aeruginosa* conveys significant anti-virulence and immunomodulatory effects, including increased cell migration and phagocytosis, the effect of *P. aeruginosa* secreted factors and NaSa on host defense was further evaluated *in vivo.* The soft tissue model included surgically-created subcutaneous pockets, in the absence or presence of titanium implants (i.e., mimicking sham wound healing and wound healing around biomaterials, respectively) (**Figure 1C**). At 24 h two distinct morphologies of the pockets were observed: open pocket (**Figure 4A**) and tight/closed pocket (**Figure 4B**). *P. aeruginosa* supernatant without NaSa (WT group) resulted in the pockets being more tightly closed after 24 h in titanium-implanted sites (12 closed pockets) and sham (21 closed pockets) compared with NaSa alone (1-5 closed pockets) or the diluent control (0-5 closed pockets) (**Figures 4C, D**). The NaSa-treated *P. aeruginosa* supernatant appeared to reduce this effect to levels comparable with the control groups. However, the NaSa-treated supernatant resulted in significantly fewer closed pockets (10) than untreated supernatants (21) at sham sites (**Figure 4C**).

The histological observations indicated that all pockets, irrespective of the presence or absence of implants, were demarcated by a vascularized loose connective tissue (**Supplementary Figure S1**; area outlined with a green dashed line) that underlined the subcutaneous musculature. Tissues that interfaced the pocket space displayed a morphology distinct from tissues that were distant from the interfacial zone.

**Figure 5:**
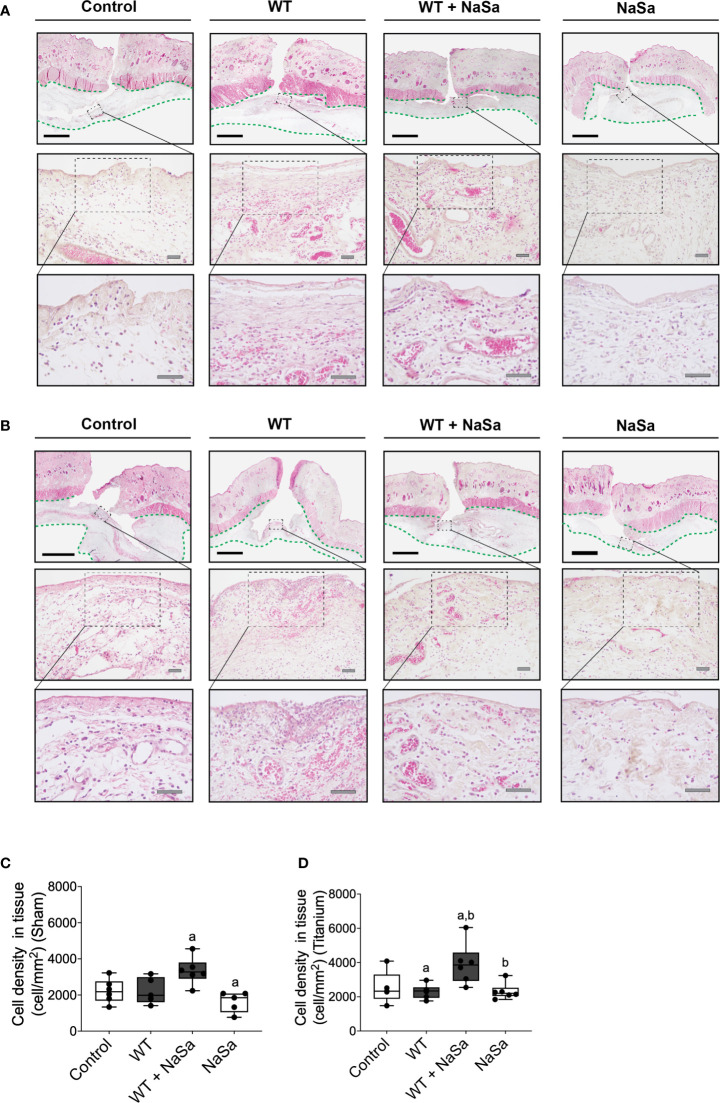
Bacterial stimuli and NaSa in both sham and titanium implantation pockets elicit morphological changes and alter the cellular infiltration in wounded soft tissues of rats. **(A, B)** Histological evaluation of the soft tissues in **(A)** sham wounds or **(B)** titanium-implanted sites at 24 h postoperative. In the loose connective tissue (delineated with a green dashed line) underneath the subcutaneous musculature, infiltrating leukocytes, which were surrounded by a proteinaceous material and blood vessels, were evident in the interfacial zone (magnified areas). Scale A & B: black=2 mm; grey=50 μm. **(C, D)** The inflammatory infiltration of soft tissues was compared between groups by morphometrically quantifying the cellular density per tissue area in histological sections from **(C)** sham wounds or from **(D)** titanium-implanted wounds at 24 h postoperative (n=5-6/group). Significant differences between treatments are indicated by letters, where bars that share the same letters are significantly different. (α) significant differences between titanium and sham (*p* < 0.05).

At the interface, while bleeding and relatively well-organized blood clots were occasionally observed, a consistent feature was the presence of prominent inflammatory infiltrate. Leukocytes had a spherical shape and consisted of mononuclear cells and polymorphonuclear leukocytes (PMNs). The extracellular material was composed of dense protein precipitates suggestive of a fibrinous material. There were fewer inflammatory cells further from the interface. These consisted mostly of mononuclear cells while PMNs were less commonly encountered (**Figures 5A, B**).

Histomorphometric analysis of the interfacial tissues demonstrated that NaSa-treated supernatants resulted in the highest density of infiltrating cells per tissue area at both titanium-implanted sites and sham sites. However, results with NaSa treatment alone, in sham or in titanium implanted sites, were comparable with the control group (**Figures 5C, D**).

In summary, supernatant from NaSa-treated *P. aeruginosa* appeared to decrease the initial fibrotic closure of soft tissue pockets and promotes the immune cell tissue influx during the early stage of wound healing, in the presence and absence of a titanium implant.

#### 3.3.1 Treatment with NaSa increases immune cell infiltration and attachment to titanium discs without affecting host cell viability

**Figure 6:**
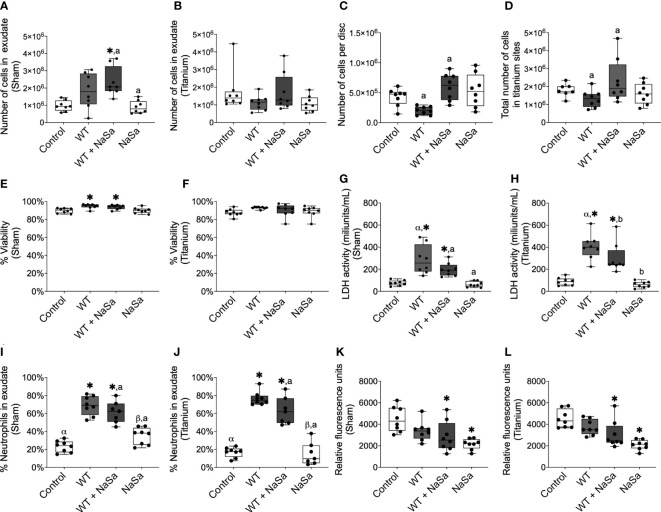
*Pseudomonas aeruginosa* supernatant influences cell recruitment and PMN percentage and reduces phagocytosis without affecting cell viability. **(A–D)** Number of cells in exudate collected from **(A)** sham and **(B)** titanium sites. **(C)** Number of cells adhered to titanium-implanted sites. **(D)** Total number of cells in titanium-implanted sites. **(E–H)** Cell viability was measured by NucleoCounter^®^ in exudate from **(E)** sham and **(F)** titanium-implanted sites and by lactate dehydrogenase (LDH) activity in the exudate from **(G)** sham and **(H)** titanium-implanted sites. **(I, J)** Percentage of polymorphonuclear cells in exudate from **(I)** sham and **(J)** titanium-implanted sites. **(K, L)** Fluorescent intensity of pH-rodo red *Staphylococcus aureus* bioparticles after 2 h phagocytosis *ex vivo* by cells collected from rat exudate from **(K)** sham and **(L)** titanium-implanted sites. Significant differences between treatments are indicated by letters, where bars that share the same letters are significantly different (*p* < 0.05), n=8. (*) significant compared to the diluent control. (α) significant between titanium and sham.

We next asked whether the secreted bacterial virulence factors may have an impact on the immune cell infiltration in the exudates of the surgical wound (sham site) and around the implanted titanium. The exudate from sham sites treated with NaSa-containing bacterial supernatant had significantly higher (2.5-fold) cell influx than the diluent and NaSa controls (**Figure 6A**). In the implantation group, the NaSa-treated supernatant resulted in significantly higher total cell recruitment at the titanium implant surface and within the exudate, as compared with the untreated supernatant (**Figures 6B–D**). In this case, the higher number of recruited cells in response to the NaSa-treated supernatant, compared with the untreated supernatant, was statistically significant for the disc-adherent cells (**Figure 6C**), but not those within the exudate around the implant (**Figure 6B**).

The viability of exudate cells was approximately 90% in all groups based on the NucleoCounter measurements (**Figures 6E, F**), however, in exudates, the treated and untreated bacterial supernatant groups showed increased cell viability compared with the diluent control (**Figure 6E**). In contrast, LDH activity was 2.6-3.8- and 3.4-4.6-fold higher in the two supernatant groups than in the control groups for the sham and titanium-implanted sites, respectively (**Figures 6G, H**). Interestingly, for the untreated supernatants, LDH activity in the exudate around titanium discs was significantly higher (≈400 mU/mL) than the exudate from sham sites (≈225 mU/mL) (**Figures 6G, H**). On the other hand, for NaSa-treated supernatants, LDH activity in the exudate around the titanium discs was not significantly different compared with that around the sham sites (250 vs 200 mU/mL) (**Figures 6G, H**).

The ratio between PMNs and mononuclear cells was increased 2.3-3.0- and 3.3-4.6-fold, in sham and titanium-implanted sites, respectively, with bacterial supernatants compared with the diluent control; no differences were observed between NaSa-treated and untreated supernatants (**Figures 6I, J**). PMN/mononuclear cell ratios were 2.7- and 1.4-fold higher in sham sites compared with titanium-implanted sites for NaSa and diluent controls, respectively (**Figures 6I, J**). Altogether, both the presence of an implant *per se*, and the inclusion of treated and untreated supernatants appear to affect the type, amount and viability of cells infiltrated in the exudate and adherent to the implant surface.

#### 3.3.2 *Ex vivo* phagocytosis is decreased by the administration of NaSa and NaSa-treated supernatants

To specifically investigate the contribution of the NaSa anti-virulence effect on phagocytosis *ex-vivo*, rat immune cells were collected and stimulated with the untreated and NaSa-treated bacterial supernatants. The phagocytic ability of cells isolated from exudates of the sham sites and titanium-implanted sites was decreased by 32-54% when stimulated with NaSa-treated supernatants or NaSa alone as compared with the diluent control (**Figures 6K, L**). No significant differences were observed between cells from sham sites and those from titanium-implanted sites. An example micrograph of pH-rodo red *S. aureus* bioparticles phagocytosed by rat immune cells is shown in **Supplementary Figure S5**. In contrast to the *in vitro* findings, supernatant from NaSa-treated *P. aeruginosa* was found to reduce the *ex vivo* phagocytic activity of the early-recruited immune cells in sham as well as in titanium implantation sites.

#### 3.3.3 *Pseudomonas aeruginosa* supernatant increases secretion of pro-inflammatory cytokines

**Figure 7:**
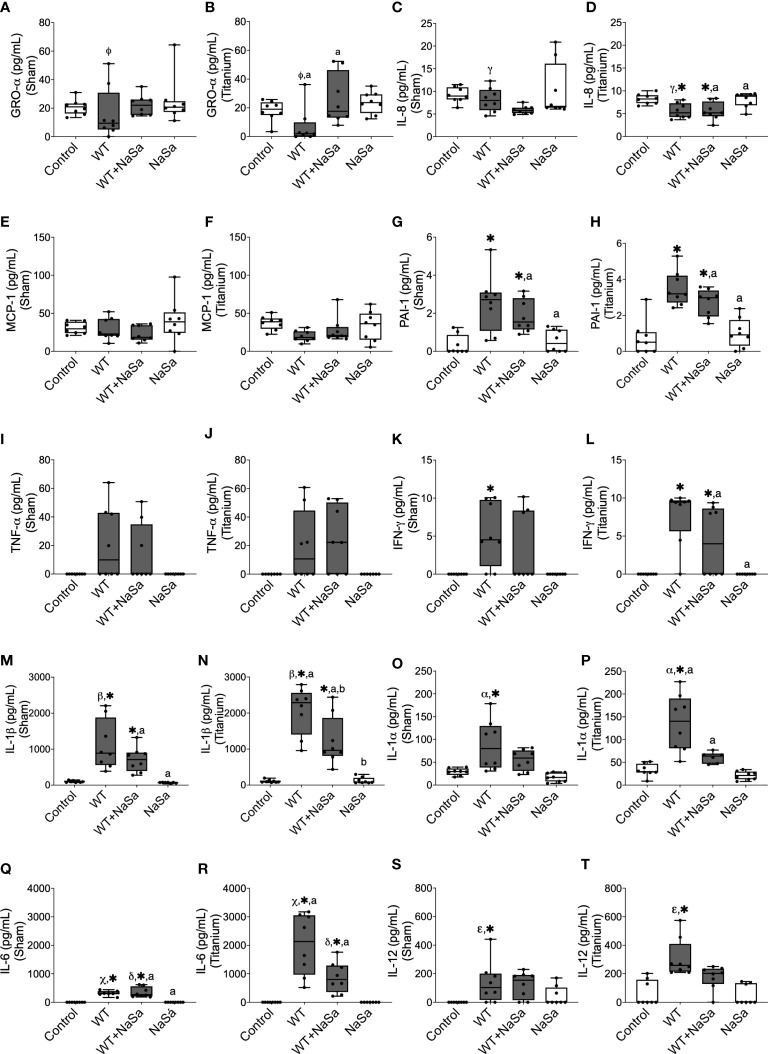
Bacterial stimuli and presence of titanium discs influence rat cytokine secretion. Secretion of **(A, B)** GRO-α, **(C, D)** IL-8, **(E, F)** MCP-1, **(G, H)** PAI-1, **(I, J)** TNF-α, **(K, L)**, IFN-γ, **(M, N)** IL-1β, **(O, P)** IL-1α, **(Q, R)** IL-6, and **(S, T)** IL-12 in sham and titanium-implanted sites. Significant differences between treatments are indicated by letters, where bars that share the same letters are significantly different (*p* < 0.05). (*) significant compared to the control. (α, β, γ, Φ, ε, χ) significant between titanium and sham (*p* < 0.05), n=8.

To explore possible mechanisms involved in the observed *in vivo* effects of the NaSa-treated bacterial supernatants, the secretion of proteins related to inflammation, infection, and tissue healing was evaluated using ELISA (**Figures 7A–T**). When comparing NaSa-treated versus untreated *P. aeruginosa* supernatants, in the titanium-implanted sites, NaSa-treatment increased the level of GRO-α (**Figure 7B**) but decreased the levels of IL-1β (**Figure 7N**), IL-1α (**Figure 7P**), and IL-6 (**Figure 7R**). Furthermore, stimulation with *P. aeruginosa* supernatants (+/- NaSa) resulted in increased levels of PAI-1 (**Figures 7G, H**), TNF-α (**Figures 7I, J**), IFN-γ (**Figures 7K, L**), IL-1β (**Figures 7M, N**), IL-1α (**Figures 7O, P**), IL-6 (**Figures 7Q, R**), and IL-12 (**Figures 7S, T**) compared with NaSa and diluent controls. Cytokine concentrations were similar between the NaSa control and the diluent control. Of the 14 different cytokines analyzed, IL-2, IL-4, and IL-10 were undetectable in all groups.

When comparing titanium-implanted sites versus sham sites, no significant differences were observed for any of the analyzed cytokines in the control groups (diluent and NaSa) (**Figures 7A–T**). Regarding the untreated supernatant, the titanium sites revealed significantly lower secretions of GRO-α (**Figures 7A, B**) and IL-8 (**Figures 7C, D**) in parallel with significantly higher secretions of IL-1β (**Figures 7M, N**), IL-1α (**Figures 7O, P**), IL-6 (**Figures 7Q, R**), and IL-12 (**Figures 7S, T**). Regarding the NaSa-treated supernatant, all cytokine differences between the titanium-implanted and sham sites disappeared with the exception of IL-6, which remained significantly higher in the titanium-implanted sites (**Figures 7Q, R**).

To summarize, stimulation with bacterial supernatants generated an inflammatory response, which was dampened by NaSa treatment. Titanium sites stimulated with bacterial supernatants although revealed lower secretion of some chemokines, they reflected higher production of major pro-inflammatory cytokines, an effect which was significantly reduced by NaSa-treatment.

#### 3.3.4 *Pseudomonas aeruginosa* supernatant modulates the gene expression of pro-inflammatory and anti-inflammatory cytokines, chemokines, and a scavenger receptor

**Figure 8:**
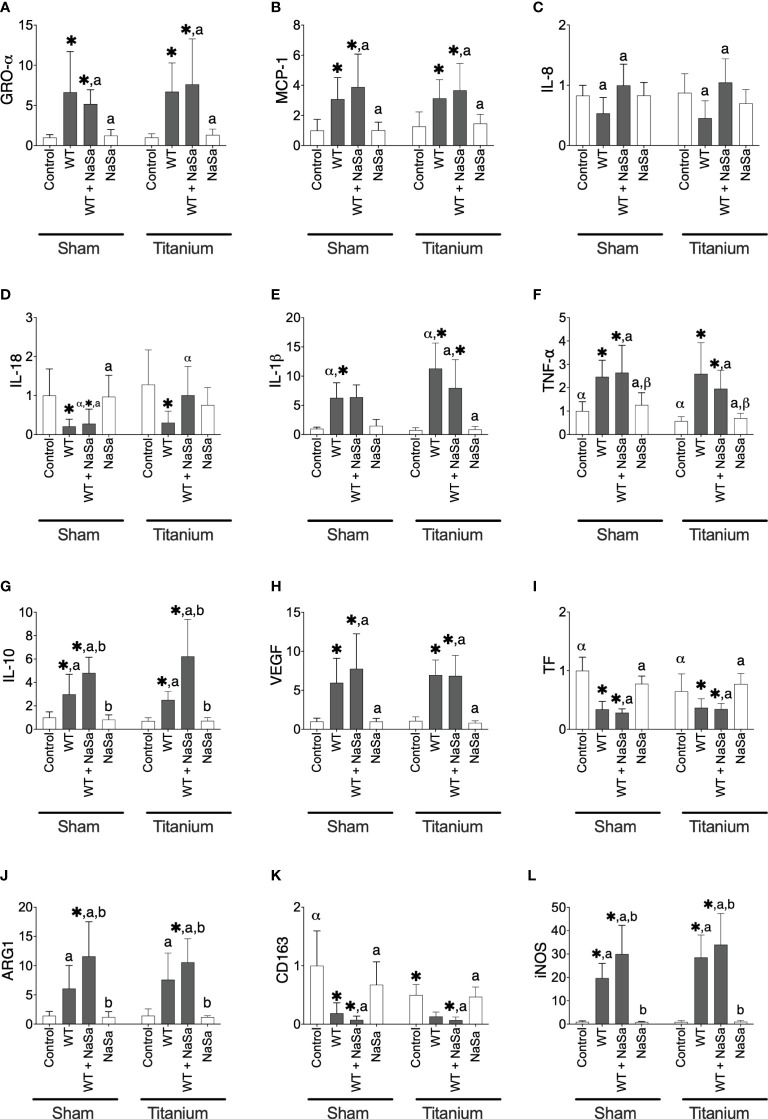
Bacterial stimuli and presence of titanium discs influence rat gene expression. Relative gene expression of **(A)** GRO-α, **(B)** MCP-1, **(C)** IL-8, **(D)** IL-18, **(E)** IL-1β, **(F)** TNF-α, **(G)** IL-10, **(H)** VEGF, **(I)** TF, **(J)** ARG1, **(K)** CD163, and **(L)** iNOS in sham and titanium-implanted sites. Significant differences between treatments are indicated by letters, where bars that share the same letters are significantly different (*p* < 0.05). (*) significant compared to the control. (α, β) significant between titanium and sham (*p* < 0.05), n=8.

To further evaluate the molecular mechanisms, gene expression analysis was performed, including genes related to inflammation, macrophage polarization and early tissue healing response. The relative gene expression of cells isolated from exudates from sham and titanium-implanted sites was evaluated by qPCR. Gene expression levels of pro-inflammatory, anti-inflammatory and coagulation proteins, chemokines, scavenger receptor and growth factors are shown in **Figure 8**. Compared with the diluent control, stimulation with both bacterial supernatants, resulted in increased levels of GRO-α, MCP-1, IL-1β, TNF-α, IL-10, VEGF, ARG1, and iNOS (**Figures 8A, B, E–H, J, L**) and decreased levels of IL-18, TF, and CD163 (**Figures 8D, I, K**). Exudates exposed to NaSa-treated supernatant, in comparison with untreated supernatants, had increased expression of the following genes: IL-8 (**Figure 8C**; 1.8-fold in sham, 2.5-fold in Ti), IL-10 (**Figure 8G**; 1.6-, 2.5-), ARG1 (**Figure 8J**; 1.9-, 1.4-), and iNOS (**Figure 8L**; 1.5-, 1.2-). PAI-1 and TGF-β1 expression levels were unchanged by the different stimuli (data not shown).

The expression of IL-18 was increased 3.8-fold in titanium-implanted sites stimulated with NaSa-treated supernatant compared to sham sites (**Figure 8D**). Levels of IL-1β were increased 1.8-fold in untreated supernatant from titanium-implanted sites compared to sham sites (**Figure 8E**). Levels of TNF-α and TF were decreased 0.6-fold and 0.9-fold, respectively, in the diluent control group from titanium-implanted sites compared to sham sites (**Figure 8F**). Taken together, these results confirmed that the stimulation with bacterial supernatants modulates the inflammatory gene expression response and demonstrated that NaSa treatment up-regulates certain genes involved in the regulation of inflammation and protection against host tissue damage. The increased expression of IL-8, iNOS, IL-10 and ARG1 in the NaSa group indicates the promotion of both M1 and M2 macrophage phenotypes at this early stage of wound healing around the titanium implants.

## 4 Discussion

Quorum-sensing (QS) inhibition has been proposed as an attractive anti-infection strategy in times when traditional antibiotics are less effective, and it is also compelling from a host response perspective. Several important pathogens utilize QS-derived signals and products to limit host defense functions and increase cell toxicity (Tateda et al., 2003; Jiajie et al., 2020). Accordingly, in animal models, infection with *P. aeruginosa* WT strains cause more harm and result in increased mortality compared with *P. aeruginosa* QS mutants (Wu et al., 2001; Lesprit et al., 2003; Kumar et al., 2009). Here, we show that supernatants from *P. aeruginosa* cultures grown in the presence of the QS inhibitor (QSI) sodium salicylate (NaSa) at a concentration of 10 mM contain lower levels of pyocyanin and the two QS signals 3-oxo-C_12_-HSL and PQS, while C_4_-HSL was unaffected. We previously showed that 0.3-10 mM NaSa, in a concentration-dependent manner, reduced the activity of the las and PQS QS systems, which respond to 3-oxo-C_12_-HSL and PQS, respectively, whereas in line with the findings of this study, the C_4_-HSL system was less affected (Gerner et al., 2020). Due to this QS-inhibitory effect, NaSa significantly decreased important virulence factors, such as pyoverdine, alkaline proteases, elastase and biofilm formation in *P. aeruginosa*.

Supernatants from *P. aeruginosa* cultured with 10 mM NaSa promoted *in vitro* migration of neutrophil-like HL-60 cells at a level similar to that induced by the potent chemokine fMLP. Interestingly, the exposure to untreated supernatants did not alter the migration capacity of the HL-60 cells. Further, the migration capacity was dependent on the concentration of bacterial stimuli, where treatment with 10% diluted *P. aeruginosa* supernatant did not affect migration. Taken together, these observations suggest that the enhancement of cell migration is due to reciprocal interactions between the HL-60 cells, specific concentration of bacterial stimuli, and the presence of NaSa. Although outside the scope of this work, NaSa may affect the bacteria and secreted factors via QS-independent mechanisms, as indicated by the significant differences in cell migration between cells subjected to NaSa-treated WT and untreated mutant supernatants, as well as between cells subjected to NaSa-treated and untreated mutant supernatants. It has previously been shown that freshly isolated human PMNs do not migrate towards undiluted PAO1 WT supernatant, whereas no such inhibitory effect was observed for QS mutants (Jensen et al., 2007). This observation is partly supported by the findings here, where the QS mutant resulted in significantly more migrating cells compared with the WT strain.

Similarly, *in vivo* administration of NaSa-treated (10 mM), but not untreated, supernatant resulted in increased cell infiltration in sham and titanium-implanted sites (predominance of PMNs). This finding could be linked to the increased GRO-α level in NaSa-treated titanium-implanted sites and IL-8 expression in NaSa-treated sham and titanium sites, as both GRO-α and IL-8 are key-players involved in neutrophil activation and recruitment during infection (Harada et al., 1994). Yet there were no major differences in GRO-α gene expression or IL-8 protein secretion when comparing the effect of NaSa-treated supernatant to untreated supernatant in sham or titanium-implanted sites. The exact reason for this discrepancy between gene expression and protein production could be attributed to multiple factors, including potential protein degradation and/or differential gene transcription and translational regulation (Greenbaum et al., 2003). Moreover, the temporal pattern of gene expression and protein production (e.g., of GRO-α and IL-8) might be dissimilar over the initial and early healing periods after implantation. Thus, the one-time point used in the study is a limitation. Taken together, the present *in vitro* and *in vivo* findings suggest that the inclusion of NaSa in an inflammatory milieu, herein provoked by *P. aeruginosa* products, promotes the migration and accumulation of inflammatory cells, particularly PMNs. This effect *in vivo* was rather linked to the presence of an implanted biomaterial like titanium.

Based on the observed effects of NaSa on immune cell migration and accumulation at sites with bacterial virulence factors simulating a local infection, it can be speculated that a treatment including NaSa could facilitate bacterial clearance via increased immune cell numbers, although the precise mechanisms and specific kinetics need to be further investigated. The present data support this idea since *in vitro* phagocytosis decreased in cells stimulated with both bacterial supernatants compared to the media control. However, NaSa-treated supernatants significantly increased phagocytosis versus untreated supernatants. After 24 h of stimulation, the phagocytic activity was 2-fold higher in cells receiving 10% NaSa-treated supernatant than in those receiving 10% untreated supernatant, with similar effects at 6 and 48 h. This is promising since it is established that phagocytosis is a pivotal immune function during infection (Rosales and Uribe-Querol, 2017) and impaired phagocytosis is associated with critical illnesses (Engelich et al., 2001).

Pyocyanin is a QS-regulated virulence factor with multiple effects on immune cells, including reducing pro-inflammatory cytokines (Marreiro De Sales-Neto et al., 2019), increasing reactive oxygen species (ROS) and hydrogen peroxide production (Hassan and Fridovich, 1980; O'malley et al., 2004), and inducing cell death (Allen et al., 2005). The supernatant from NaSa-treated *P. aeruginosa* contained approximately 7-fold less pyocyanin than the untreated supernatant. Although not evaluated in this study, excessive ROS production (e.g., induced by high pyocyanin levels) causes tissue destruction (Bergamini et al., 2004), can attenuate macrophage function including phagocytosis (Anderson et al., 2002; De Groot et al., 2019), and might have contributed to the reduced phagocytic capability found in the presence of untreated supernatants. Furthermore, the *P. aeruginosa* type-III secretion system (T3SS) is capable of injecting effector proteins and toxins into host cells, which have been shown to reduce macrophage uptake of *P. aeruginosa* (Lovewell et al., 2014). The T3SS is under QS regulation (Pena et al., 2019) and is yet another possible mechanism that could contribute to the observed differences in phagocytosis. However, QS-related factors do not only attenuate immune functions. For instance, it has been shown that 3-oxo-C_12_-HSL promotes phagocytosis in human macrophages (Vikström et al., 2005) and that PAO1 WT cells are more readily phagocytosed than QS mutant cells (Holm et al., 2015). Furthermore, 3-oxo-C_12_-HSL and PQS can stimulate epithelial (Karlsson et al., 2012) and PMN (Hansch et al., 2014) cell migration. The observed variation can be explained by the potential effects of factors such as culture age (planktonic or biofilm stage) (Ciszek-Lenda et al., 2019), presence of serum components (Kruczek et al., 2014), QS signal concentration (Tateda et al., 2003), and evaluation of QS signals alone or in combination with bacterial stimuli (Jiajie et al., 2020). These findings highlight the complex role of QS inhibition and its effect on immune functionality.

The NaSa control resulted in a clear reduction in phagocytosis compared to the diluent control in cells from both the titanium-implanted and sham sites, whereas contrary to the *in vitro* results, no difference was observed between NaSa-treated (10 mM) and untreated supernatants *in vivo*. NaSa (1 mM) and acetylsalicylic acid (≥ 5 mM) have previously been shown to suppress phagocytosis in rabbit PMNs (Chang, 1972) and mouse macrophages (Cai et al., 2011), respectively. In the latter study, acetylsalicylic acid concentrations above 3 mM were required to reduce phagocytosis. Hence, the discrepancy between the *in vitro* and *in vivo* NaSa control data could be a result of concentration dissimilarities, as NaSa concentrations used were 5-fold lower *in vitro* (2 mM) than *in vivo* (10 mM), due to the *in vitro* cell viability constraints with the bacterial supernatants. In the present *in vivo* study, a single administration of stimuli was used, but pharmacokinetics and bioavailability were not studied, making concentration-related conclusions not possible. Clinically, salicylate concentrations of up to 300 mg/L (2.2 mM) in blood are used therapeutically, whereas higher concentrations are considered toxic (Pearlman and Gambhir, 2009). For topical administration of over-the-counter products intended for intact skin, salicylate concentrations of approximately 5% are used (360 mM) (Madan and Levitt, 2014). Considering that the percutaneous absorption of salicylic acid is approximately 10-60% (Schwarb et al., 1999), the highest concentration used in the present study (10 mM) is within the safe range. However, it is important to recognize that the safety of NaSa administration in a clinical setting, such as for wound treatment, will be highly dependent on factors such as dosing intervals, wound size and rate of clearance.

*Pseudomonas aeruginosa* produces several harmful products, such as rhamnolipids (Jiang et al., 2014), pyocyanin (Hall et al., 2016), and the T3SS (Galle et al., 2012). Further, cell-free supernatants from *P. aeruginosa* have proven to be cytotoxic (Lee et al., 2011; Shinagawa et al., 2014). Although cell viability in this study was clearly decreased by bacterial supernatants *in vitro*, regardless of the inclusion of NaSa-treatment, cell viability was overall high and similar between all groups *in vivo*. Exudate cells stimulated with untreated bacterial supernatants demonstrated slightly improved viability compared with cells isolated from the diluent control. However, direct comparison of cell toxicity *in vivo* is challenging. The relative proportions of PMNs and mononuclear cells differed markedly between the supernatant-treated and control groups, which may account for differences in observed viability. Interestingly, the presence of titanium discs resulted in a lower PMN/mononuclear cell ratio in both control groups, which was also shown previously in the same animal model 4 h postsurgery (Svensson et al., 2015). Inflammation and regeneration at the implant-tissue interface are mediated by host intercellular communication. For example, monocytes/macrophages at implant sites communicate with mesenchymal stem cells, which are important in the healing and regenerative processes, and affect their differentiation (Ekstrom et al., 2013). Intriguingly, exudates from both *P. aeruginosa* supernatants (+/- NaSa) showed a decrease in the gene expression of the scavenger receptor CD163, which functions as an innate immune sensor for bacteria and has a key role in host defence (Fabriek et al., 2009). It has been reported that during infection, CD163 triggers the production of pro-inflammatory cytokines. However, as negative feedback, CD163 expression is down-regulated by pro-inflammatory cytokines, including TNF-α and IL-1β (Zhu et al., 2020). The presence of LPS in the supernatant may also explain the downregulation of CD163 expression *in vivo*. LPS induces NF-κB activity, which elicits the release of pro-inflammatory cytokines. This produces an increase in the expression of disintegrin and metalloproteinase 17 (ADAM17), which is directly involved in the downregulation of CD163 (Zhu et al., 2020). Since CD163 is highly expressed in phagocytic macrophages, the lack of expression observed in exudates may explain the relative reduction in phagocytosis with *P. aeruginosa* supernatants cultured ± NaSa compared to the diluent control.

While animals stimulated with bacterial supernatants generated a pro-inflammatory response, this response was reduced with 10 mM NaSa-treated supernatant, as the secretion levels of IL-1β, IL-6, and IL-1α were significantly reduced compared with those in animals administrated untreated supernatant. Previously, NaSa (2-20 mM) has been shown to possess anti-inflammatory properties and reduce LPS-triggered NF-κB activity *in vitro* (Kopp and Ghosh, 1994). NF-κB is a transcription factor activated via, e.g., LPS-TLR4 interaction, with promoting effects on pro-inflammatory factors such as IL-1, IL-6, IL-12, and TNF-α in macrophages (Wang et al., 2014). Among the studied cytokines, IL-6 showed the strongest decrease by the NaSa-treated supernatant compared with the untreated supernatant. IL-6 is associated with chronic inflammation (Gabay, 2006), and has been suggested as a biomarker of infection. Moreover, IL-6 has been reported to be elevated in chronic wounds (Kishimoto et al., 1992) and in the synovial fluid from periprosthetic joint infections (PJIs) (Lee et al., 2017). Similarly, IL-1β, which was the second most decreased cytokine by the NaSa-treated supernatant compared with the untreated supernatant, has been found to be elevated in both chronic wound fluid (Tarnuzzer and Schultz, 1996) and PJI synovial fluid (Wang et al., 2021). Indeed, excessive or abnormal inflammation is associated with delayed healing and tissue destruction (Raziyeva et al., 2021). The use of QSIs against *P. aeruginosa* has previously been shown to reduce pro-inflammatory cytokines. For example, baicalein treatment of *P. aeruginosa* has been shown to reduce IL-1β, IL-6, IL-8, and TNF-α via the mitogen-activated protein kinase (MAPK) and NF-κB signal-transduction pathways (Luo et al., 2016). Agents which have direct or indirect anti-inflammatory effects may aid in the treatment of excessive inflammation, which could be beneficial, not only for eradicating the causative microorganism but also for the resolution of chronic wounds and biomaterial-associated infections. For instance, IL-10 is a critical anti-inflammatory cytokine that acts as a negative feedback regulator of exacerbated pro-inflammatory cytokine production (Peñaloza et al., 2018). In this study, animals stimulated with NaSa-treated *P. aeruginosa* supernatant showed upregulation of IL-10 in comparison with those stimulated with untreated supernatant. This supports the assumption that NaSa treatment protects against host tissue damage and promotes resolution of inflammation.

NaSa-dependent effects on NF-κB could have also played a role in the reduction of wound tightness observed in animals receiving NaSa-treated supernatant compared with untreated supernatant. NF-κB induces fibrin deposition and plasminogen activator inhibitor 1 (PAI-1) expression, collectively resulting in increased fibrin levels (Libby and Simon, 2001; Levi et al., 2003), which could contribute to the observed tight pockets. PAI-1 was more secreted in supernatant-stimulated animals than controls, which may play a role in the observation of tight pockets in these animals. However, PAI-1 levels did not reveal any NaSa-dependent differences at 24 h, hence, further investigations are needed to explain the difference in the number of tight pockets between NaSa-treated and untreated supernatants.

Interestingly, gene expression of inducible nitric oxidase synthase (iNOS), a hallmark of classically activated macrophages, was increased in exudates from *P. aeruginosa* supernatant cultured with and without the QSI NaSa. iNOS is involved in host defence against bacterial infection by stimulating the production of downstream proinflammatory signals and promoting the release of ROS such as nitric oxide. It is well characterized that LPS can induce the classically activated macrophage phenotype which may explain our findings (Macmicking et al., 1997). Notably, gene expression of arginase 1 (ARG1), a marker of alternatively activated macrophages, was increased by *P. aeruginosa* NaSa-treated supernatant in exudates from sham and titanium-implanted sites in comparison with untreated supernatants. ARG1 induction could be beneficial as it inhibits excessive nitric oxide production and regulates inflammation, and has been shown to be required for cutaneous wound healing (Campbell et al., 2013).

Titanium, a widely used biomaterial for bone-anchored implants (Sidambe, 2014), was employed as a model implantable device. The observed cellular and molecular responses observed at the immediate tissue milieu of our *in vivo* model concur with previous studies in verifying the early and robust chemotactic property of titanium implants (Suska et al., 2001; Suska et al., 2003). Leukocyte recruitment peaks at 24 h at the titanium implant interface (Suska et al., 2001; Gretzer et al., 2006; Svensson et al., 2015), and is driven by the release of cytokines and chemokines by inflammatory cells (Suska et al., 2003). Here, one major observation is the increased cell migration in sham and titanium-implanted sites stimulated with 10 mM NaSa-treated supernatant. This heightened cellular recruitment by the NaSa-treated supernatant, versus untreated supernatant, was demonstrated by the increased cellular presence at the interfacial soft tissue, in exudates, and on titanium discs. Based on the present findings, we postulate that NaSa-treated *P. aeruginosa* potently enhances the migration and influx of inflammatory cells to the wound site, and if the titanium implant is encountered, the migrated cells preferably adhere to its surface. From a clinical perspective, this could be beneficial in terms of infection control. In the late 1980s, Gristina and coworkers coined the term “race for the surface”, which describes the initial events occurring at the surface of an implanted biomaterial, where microorganisms and host cells will compete and interact to colonize the implant surface. Therefore, in this study, we evaluated the recruitment, adhesion/colonization, and phagocytosis processes important for host cell-bacteria interactions. In theory, fast recruitment and coverage of host cells would limit bacterial colonization and the risk of implant failure due to infection (Gristina, 1987). However, proper tissue integration can be challenging due to the hypothesized immunocompromised zone surrounding the implant, which is characterized by excessive ROS levels and tissue damage (Gristina, 1994).

## 5 Conclusion

The present study shows that treatment of *P. aeruginosa* with NaSa has a dual function: (i) an anti-virulence effect by reducing the secretion of QS signals and virulence factors, and (ii) increasing *in vitro* immune cell chemotaxis and phagocytic ability, *in vivo* immune cell influx, and reducing pro-inflammatory cytokine secretion. Taken together, these data suggest that NaSa administration could be beneficial in the clinical treatment of chronic infections with elevated inflammation, possibly reducing the need for antibiotics, although additional *in vivo* infection studies with viable bacteria and longer observation periods are needed to further evaluate this treatment concept.

## Data availability statement

The original contributions presented in the study are included in the article/**Supplementary Material**, further inquiries can be directed to the corresponding author/s.

## Ethics statement

The animal study was reviewed and approved by The Local Ethical Committee for Laboratory Animals (Dnr 1091/17) in Gothenburg (Sweden).

## Author contributions

EG and PG-O have contributed equally to this work and share first authorship. EG, PG-O, MW, AP, PT, OO, SA and MT contributed to the conception and design of the study. EG, PG-O, AJL, RF and HBA performed the experimental work. EG, PG-O, RF and HBA carried out the analysis and graphical representation of data. EG, PG-O, HBA, SA, PT, MW, OO and MT interpreted the data. EG and PG-O drafted the manuscript. AJL, RF, HBA, MW, AP, PT, OO, SA and MT critically revised the manuscript. All authors contributed to the article and approved the submitted version.
