# Supplementary material for: Targeting Pseudomonas aeruginosa quorum sensing with sodium salicylate modulates immune responses in vitro and in vivo
Source: Front Cell Infect Microbiol. 2023 Aug 8;13:1183959. doi: 10.3389/fcimb.2023.1183959 (PMC10442818; doi:10.3389/fcimb.2023.1183959)
Supplement: Supplementary file 1 [file Presentation_1.pptx]

## Slide 1
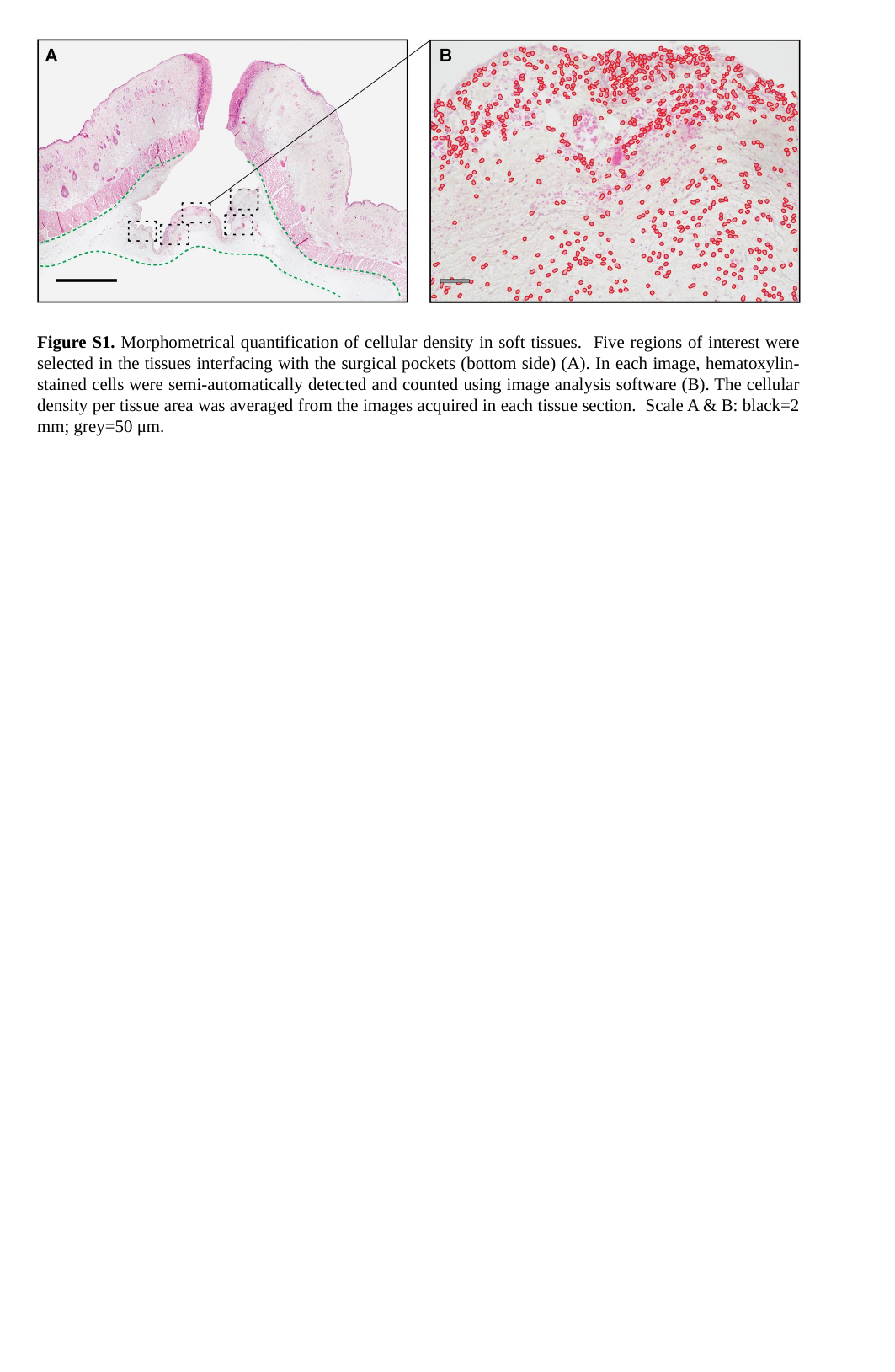

Figure S1. Morphometrical quantification of cellular density in soft tissues. Five regions of interest were selected in the tissues interfacing with the surgical pockets (bottom side) (A). In each image, hematoxylin-stained cells were semi-automatically detected and counted using image analysis software (B). The cellular density per tissue area was averaged from the images acquired in each tissue section. Scale A & B: black=2 mm; grey=50 μm.

## Slide 2
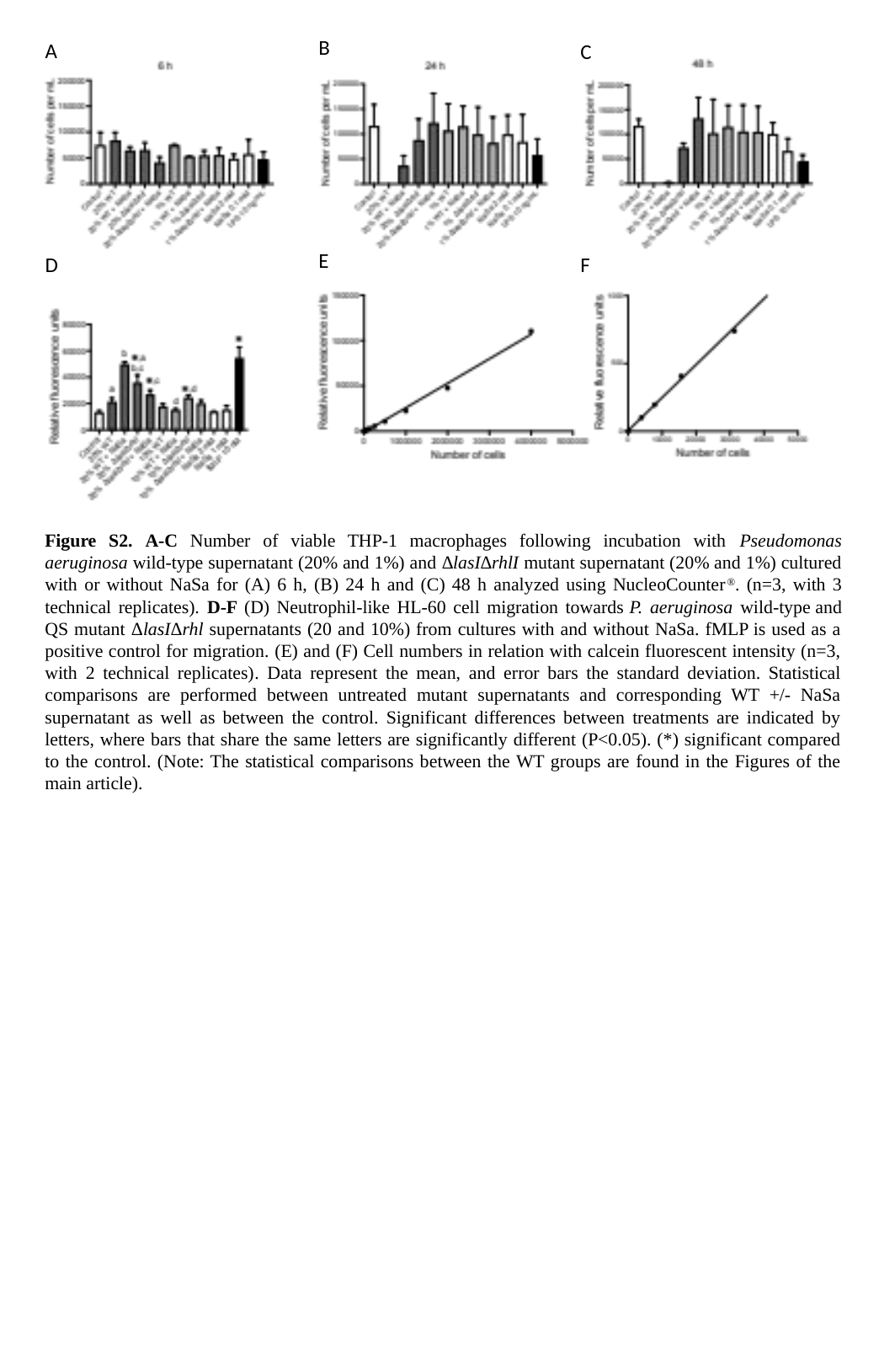

A
B
A
C
E
D
F
Figure S2. A-C Number of viable THP-1 macrophages following incubation with Pseudomonas aeruginosa wild-type supernatant (20% and 1%) and ∆lasI∆rhlI mutant supernatant (20% and 1%) cultured with or without NaSa for (A) 6 h, (B) 24 h and (C) 48 h analyzed using NucleoCounter®. (n=3, with 3 technical replicates). D-F (D) Neutrophil-like HL-60 cell migration towards P. aeruginosa wild-type and QS mutant ΔlasIΔrhl supernatants (20 and 10%) from cultures with and without NaSa. fMLP is used as a positive control for migration. (E) and (F) Cell numbers in relation with calcein fluorescent intensity (n=3, with 2 technical replicates). Data represent the mean, and error bars the standard deviation. Statistical comparisons are performed between untreated mutant supernatants and corresponding WT +/- NaSa supernatant as well as between the control. Significant differences between treatments are indicated by letters, where bars that share the same letters are significantly different (P<0.05). (*) significant compared to the control. (Note: The statistical comparisons between the WT groups are found in the Figures of the main article).

## Slide 3
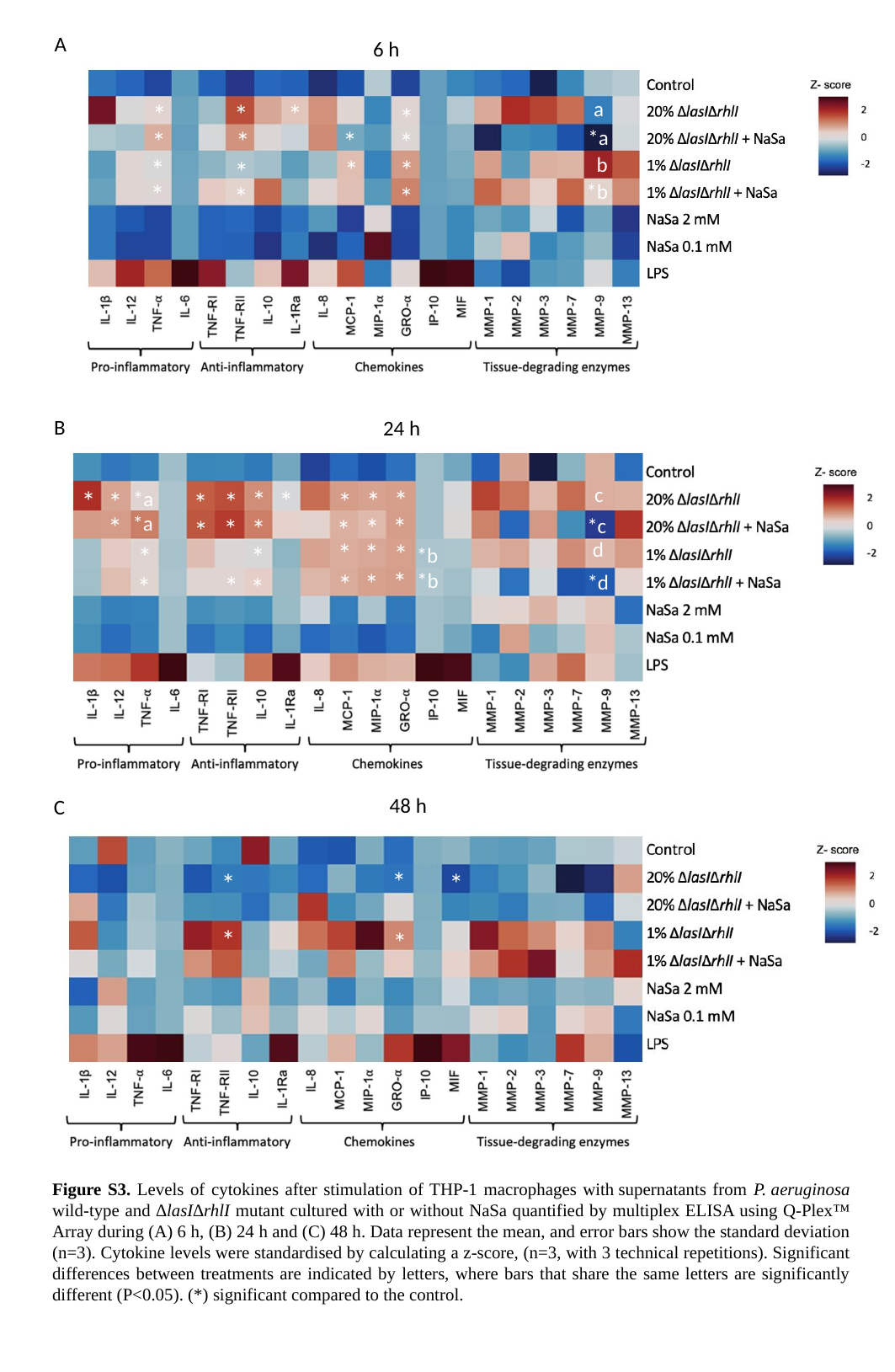

A
6 h
*
*
*
*
a
*
*
*
*
*
*a
*
b
*
*
*
*b
*
*
B
24 h
c
*
*
*
*
*a
*
*c
d
*
*
*
*d
*
*
*
*
*
*
*
*a
*
*
*
*
*
*
*
*
*b
*
*
*b
*
*
48 h
C
*
*
*
*
*
Figure S3. Levels of cytokines after stimulation of THP-1 macrophages with supernatants from P. aeruginosa wild-type and ∆lasI∆rhlI mutant cultured with or without NaSa quantified by multiplex ELISA using Q-Plex™ Array during (A) 6 h, (B) 24 h and (C) 48 h. Data represent the mean, and error bars show the standard deviation (n=3). Cytokine levels were standardised by calculating a z-score, (n=3, with 3 technical repetitions). Significant differences between treatments are indicated by letters, where bars that share the same letters are significantly different (P<0.05). (*) significant compared to the control.

## Slide 4
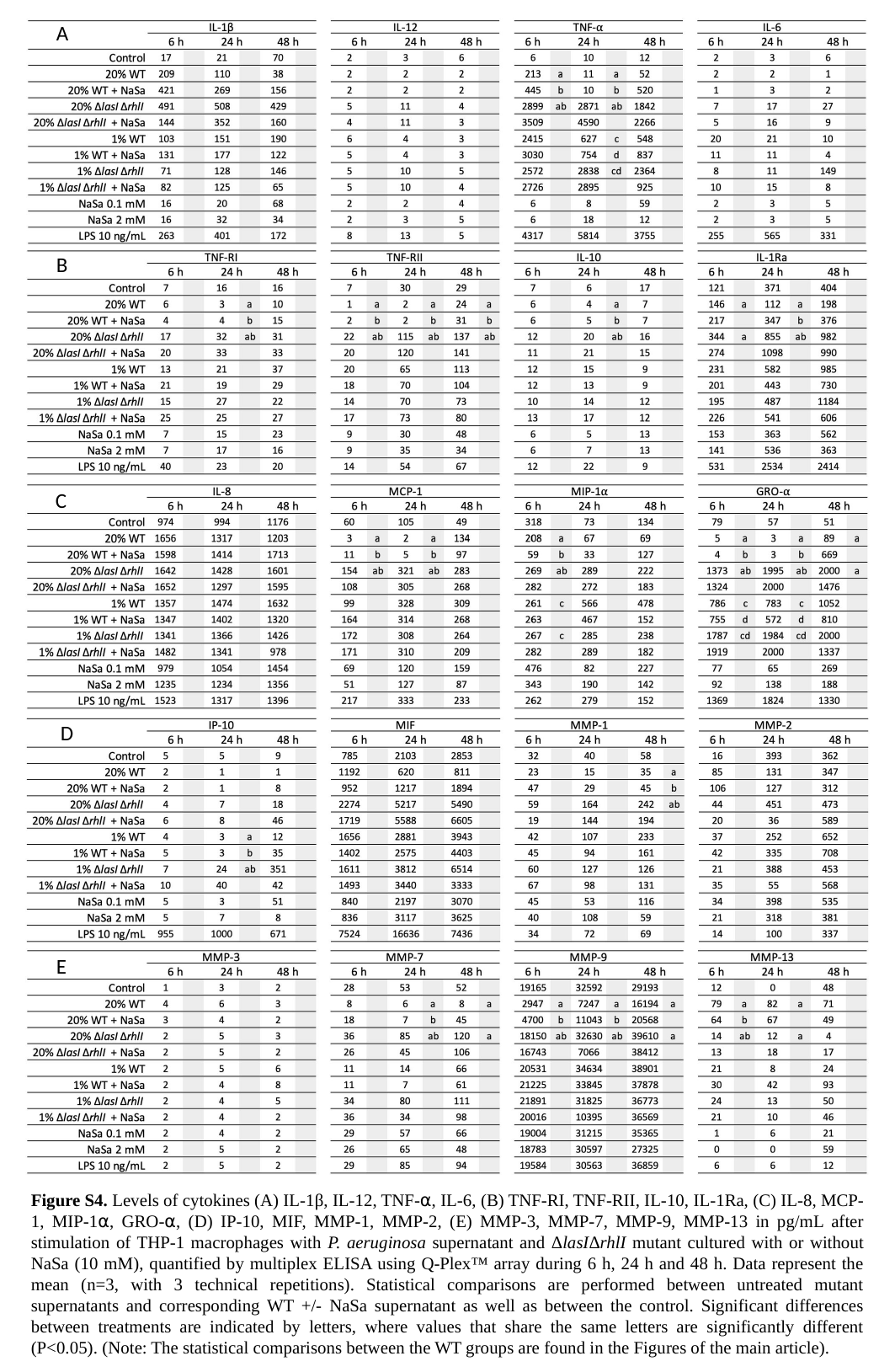

A
B
C
D
E
Figure S4. Levels of cytokines (A) IL-1β, IL-12, TNF-⍺, IL-6, (B) TNF-RI, TNF-RII, IL-10, IL-1Ra, (C) IL-8, MCP-1, MIP-1⍺, GRO-⍺, (D) IP-10, MIF, MMP-1, MMP-2, (E) MMP-3, MMP-7, MMP-9, MMP-13 in pg/mL after stimulation of THP-1 macrophages with P. aeruginosa supernatant and ΔlasIΔrhlI mutant cultured with or without NaSa (10 mM), quantified by multiplex ELISA using Q-Plex™ array during 6 h, 24 h and 48 h. Data represent the mean (n=3, with 3 technical repetitions). Statistical comparisons are performed between untreated mutant supernatants and corresponding WT +/- NaSa supernatant as well as between the control. Significant differences between treatments are indicated by letters, where values that share the same letters are significantly different (P<0.05). (Note: The statistical comparisons between the WT groups are found in the Figures of the main article).

## Slide 5
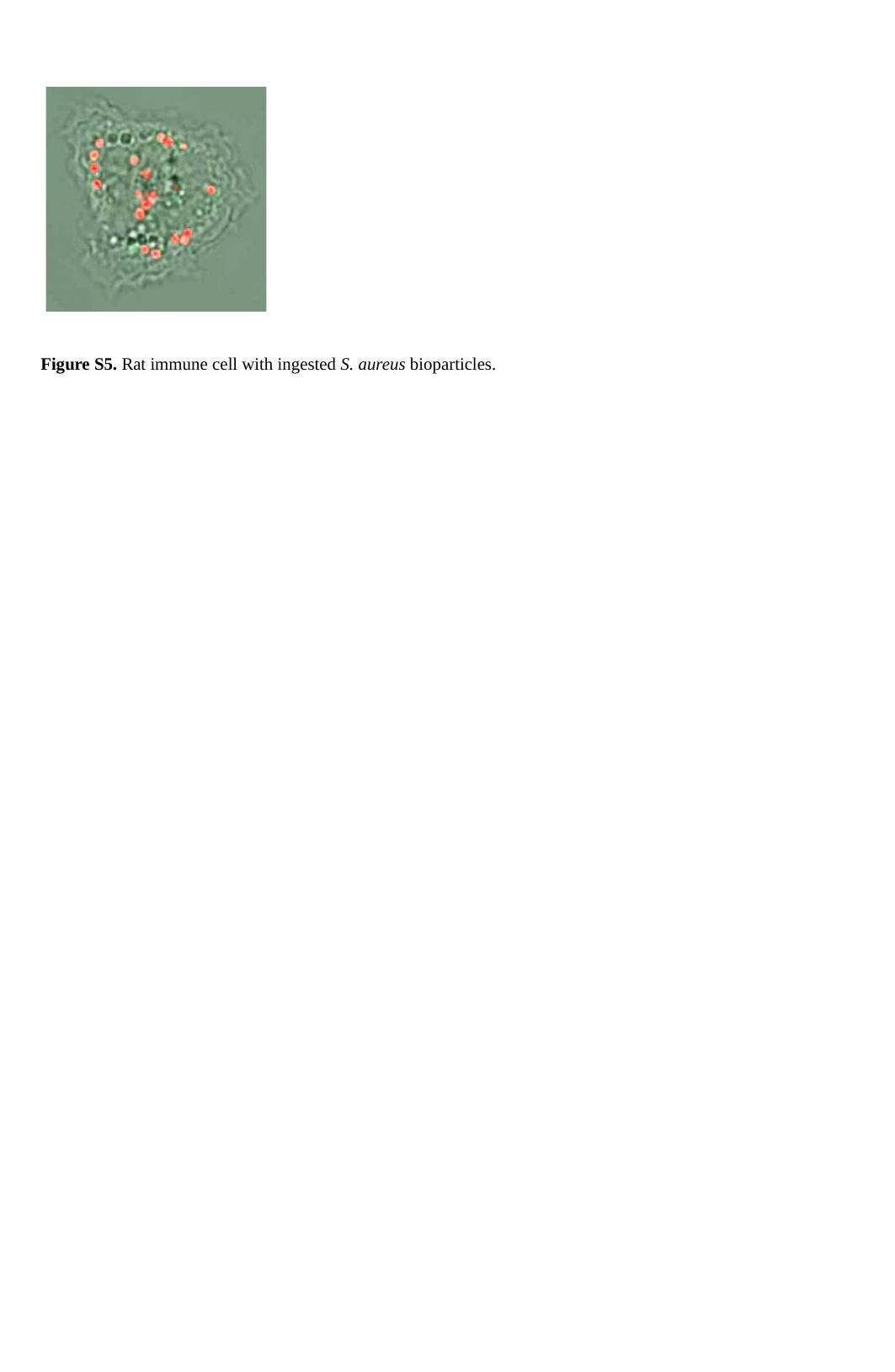

Figure S5. Rat immune cell with ingested S. aureus bioparticles.
